# Transition of *Plasmodium* Sporozoites into Liver Stage-Like Forms Is Regulated by the RNA Binding Protein Pumilio

**DOI:** 10.1371/journal.ppat.1002046

**Published:** 2011-05-19

**Authors:** Carina S. S. Gomes-Santos, Joanna Braks, Miguel Prudêncio, Céline Carret, Ana Rita Gomes, Arnab Pain, Theresa Feltwell, Shahid Khan, Andrew Waters, Chris Janse, Gunnar R. Mair, Maria M. Mota

**Affiliations:** 1 Malaria Unit, Instituto de Medicina Molecular, Lisboa, Portugal; 2 PhD Programme in Experimental Biology and Biomedicine, Center for Neuroscience and Cell Biology, University of Coimbra, Coimbra, Portugal; 3 Leiden Malaria Research Group, Parasitology, Leiden University Medical Centre, Leiden, The Netherlands; 4 Molecular Parasitology Unit, Instituto de Medicina Molecular, Lisbon, Portugal; 5 Pathogen Genetics Group, Wellcome Trust Sanger Institute, Cambridge, United Kingdom; 6 Computational Bioscience Research Center (CBRC), Chemical Life Sciences and Engineering Division, King Abdullah University of Science and Technology (KAUST), Thuwal, Kingdom of Saudi Arabia; 7 Division of Infection and Immunity, Institute of Biomedical Life Sciences and Wellcome Centre for Molecular Parasitology, Glasgow Biomedical Research Centre, University of Glasgow, Glasgow, Scotland; University of Geneva, Switzerland

## Abstract

Many eukaryotic developmental and cell fate decisions that are effected post-transcriptionally involve RNA binding proteins as regulators of translation of key mRNAs. In malaria parasites (*Plasmodium* spp.), the development of round, non-motile and replicating exo-erythrocytic liver stage forms from slender, motile and cell-cycle arrested sporozoites is believed to depend on environmental changes experienced during the transmission of the parasite from the mosquito vector to the vertebrate host. Here we identify a *Plasmodium* member of the RNA binding protein family PUF as a key regulator of this transformation. In the absence of Pumilio-2 (Puf2) sporozoites initiate EEF development inside mosquito salivary glands independently of the normal transmission-associated environmental cues. *Puf2-* sporozoites exhibit genome-wide transcriptional changes that result in loss of gliding motility, cell traversal ability and reduction in infectivity, and, moreover, trigger metamorphosis typical of early *Plasmodium* intra-hepatic development. These data demonstrate that Puf2 is a key player in regulating sporozoite developmental control, and imply that transformation of salivary gland-resident sporozoites into liver stage-like parasites is regulated by a post-transcriptional mechanism.

## Author Summary

Injection of *Plasmodium* sporozoites by *Anopheles* mosquitoes into the human host initiates malaria infection. Generation of blood stage parasites leading to the onset of disease relies on the successful development of the sporozoite into merozoites in the liver. Here we show that in the rodent malaria model *Plasmodium berghei* these developmental transformations are controlled by the RNA binding protein Pumilio 2 (Puf2). In the absence of Puf2, sporozoites, while still in the mosquito salivary gland, slowly transform into early stage exo-erythrocytic form parasites without the need for any environmental signals. Morphologically precocious metamorphosis is characterized by the loss of the inner membrane complex and the generation of clear protrusions which result in round liver stage-like parasites. The transcriptional alterations are consistent with developmental adaptations that occur during sporozoite to liver stage-like developmental progression. In total, 90% of up-regulated genes in mutant parasites have only been identified in liver stage parasite proteomes, but not the sporozoite proteome. *Plasmodium* Puf2, a member of the Pumillo family known to be involved in key developmental decisions in many different organisms, acts as a clear regulator of life cycle progression during sporozoite transmission from the mosquito vector to the mammalian host.

## Introduction

Puf (Pumilio and fem-3 mRNA binding factor) proteins are an evolutionarily highly conserved family of proteins present from yeast to humans and plants characterized by a highly conserved C-terminal RNA-binding domain, composed of eight tandem Pumilio (PUM) repeats. Puf proteins typically decrease expression of targeted mRNAs by enhancing their decay or repressing their translation [reviewed in 1]. The conserved biochemical features and genetic function of Puf family members have emerged from studies of model organisms and although Puf proteins have been shown to play diverse functions, the one frequently shared throughout evolution relates to the maintenance of stemness [2], [3], [4] and control of differentiation [5], [6], [7].

The *Plasmodium* parasite alternates between mosquito vector and vertebrate host, with transmission relying on highly specialized parasite stages. Once inside the new host, developmental progression quickly gives rise to fundamentally different parasite forms adapted to their new environment [8]. For example, cell-cycle arrested gametocytes, transmitted from the mammalian host to the *Anopheles* vector during a mosquito blood meal, fertilize and generate the motile ookinete in the mosquito midgut. Similarly, a single slender, motile and cell-cycle arrested sporozoite, transmitted by a mosquito bite, while inside a liver cell will develop into a round, non-motile and replicating exo-erythrocytic form (EEF) and go on to generate thousands of merozoites [9], [10], [11]. Developmental progression of both gametocytes and sporozoites requires clear environmental cues; for gametocytes these include xanthurenic acid and a drop in temperature [12], while sporozoites need a rise in temperature and the presence of bicarbonate [13], [14], [15].

The sudden transition between hosts that have very different physiological environments requires a rapid molecular and cellular re-programming, which may only be realized by parasites that are in a state of molecular preparedness, while maintaining a quiescent state until transmission occurs. Indeed, successful development of the mosquito-infective ookinete relies on the availability of translationally repressed mRNAs previously transcribed in female gametocytes in the blood stage, which are only translated following fertilization [16], [17], as well as stored proteins [18]. Although suggested [19], [20] it is unknown whether equivalent post-transcriptional RNA-mediated events facilitate developmental progression during the parasite's exit from the mosquito and initiation of EEF development in the mammalian host liver. Still, *Plasmodium* sporozoites remain viable and transmission-competent for weeks in mosquito salivary glands [21].

The roles of Puf (Pumilio and fem-3 mRNA binding factor) proteins are diverse yet intimately involved in the post-transcriptional regulation of developmental and differentiation factors in organisms as diverse as yeast, *Caenorhabdites elegans*, *Drosophila* and humans [22], [23], [24], [25]. Two such proteins, Puf1 (PFE0935c) and Puf2 (PFD0825c), are known in the human malaria parasite *P. falciparum* [26], [27], [28], with orthologs in all *Plasmodium* species characterized, including the rodent-infectious species *P. berghei*. The *Plasmodium* Puf proteins have the typical highly conserved organization that includes the eight tandem copies of the PUM RNA binding domain (or Pumilio homology domain, PHD) at the carboxyterminus of the protein (Fig. S1) and *P. falciparum* Puf2 was shown to bind the Nanos Response Element RNA *in vitro* [26]. In *P. falciparum* evidence has been reported for a role for Puf2 in gametocyte development although *puf2* is most highly transcribed in sporozoites [28], [29].

Here we provide strong evidence for an RNA-mediated regulatory event in the rodent malaria parasite *P. berghei* that relies on the RNA binding protein Pumilio 2 (PBANKA_071920) to maintain salivary gland sporozoites in a stand-by mode prior to transmission. The absence of the highly conserved protein Pumilio-2 is necessary and sufficient to enable the slow and progressive morphological transformation of *P. berghei* sporozoites into EEF-like forms while still inside the lumen of the mosquito salivary gland. This transformation is characteristic of EEFs both functionally and in respect to their gene expression repertoire and dissociates the transformation of sporozoites to EEF-like forms from its requirement for environmental cues.

## Results

### *puf2^-^ P. berghei* sporozoites undergo EEF-like metamorphosis inside mosquito salivary glands

**Figure 1:**
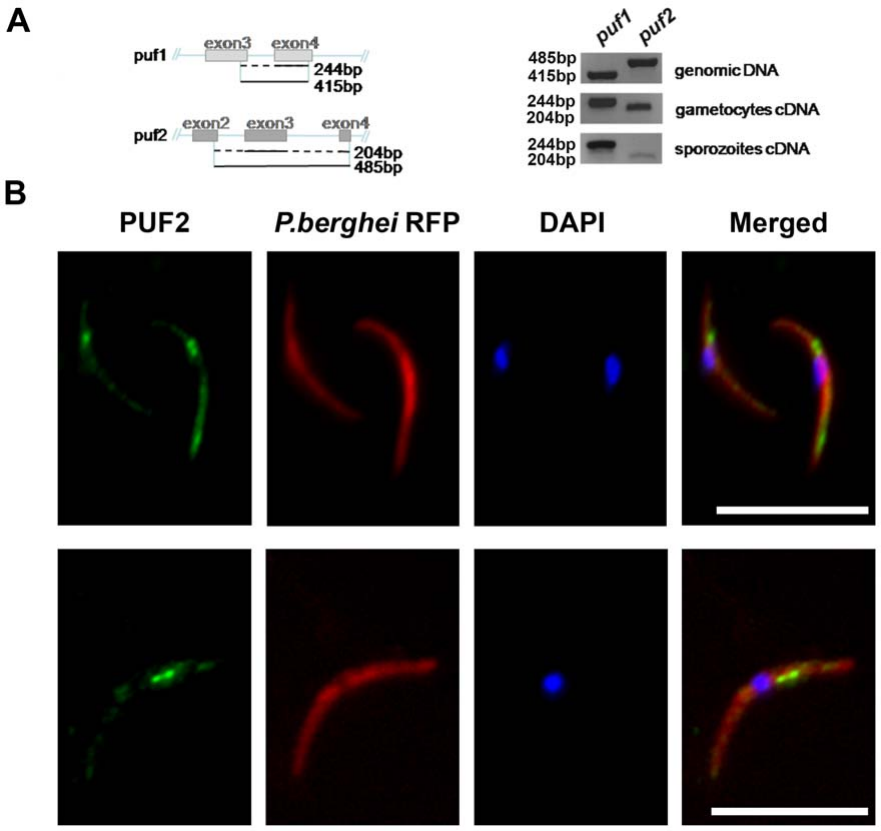
*puf* mRNA expression and immunolocalization in *P. berghei* RFP+ salivary gland sporozoites. **A**, Relative and stage-specific transcription levels of *puf1* and *puf2* in wild type parasites. *puf1* and *puf2* steady state mRNA levels were analyzed by RT-PCR using cDNA from highly purified gametocytes and day 18 salivary gland (SG) sporozoites. Genomic DNA amplification is also provided, together with a map showing the localisation of primers used for amplifying *puf1* and *puf2* and the different sizes to be expected in cDNA and genomic DNA. See also Fig. S3C for Northern analysis of *puf2* throughout parasite life cycle. **B,** Sporozoites tagged with red fluorescent protein (line 733cl1) were stained with anti-Puf2 peptide antibody 904 (top panel) and 905 (lower panel) revealing distinct cytoplasmic protein speckles. Scale bars = 10 µm.

Similar to P. falciparum, both *P. berghei puf* orthologs (*puf1*, PBANKA_123350 *and puf2,* PBANKA_071920) are not only transcribed in gametocytes but also in salivary gland sporozoites (SGS) (Fig. 1A, S3C). Antibodies raised against PbPuf2 confirmed the expression of the protein in SGS and localized the protein to a small number of discrete foci in the cytoplasm of the cell consistent with the localization of most Puf proteins (Fig. 1B). To address the roles of the two encoded proteins during parasite transmission we generated transgenic *P. berghei* that lack either *puf1* or *puf2*, or both genes (Figs. S2–S4). All 3 gene deletion mutants (*puf1*-, *puf2-*, *puf1*-/2-) showed normal growth and multiplication of asexual blood stage parasites and in contrast to the reports for *P. falciparum* produced gametocytes comparable in number to wild type parasites; the transition of gametocytes into gametes and ookinete formation was also not affected (Table S1). Furthermore oocyst numbers per midgut and sporozoites reaching the salivary glands were not significantly altered when compared to wild type parasites (Fig. S5).

**Figure 2:**
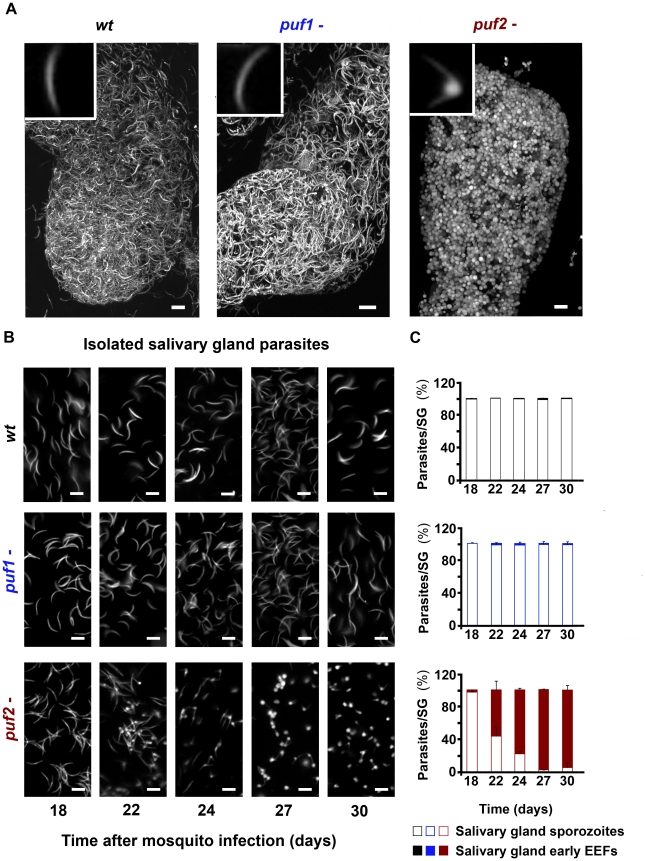
*puf2-* parasites transform into early liver stage EEF's in mosquito salivary glands. **A**, maximum projection of a z-series scan of infected mosquito salivary glands on day 30 of mosquito infection. **B**, Development of salivary gland-resident parasites from day 18 to day 30 after mosquito infection; *puf2-* undergo exo-erythrocytic liver stage metamorphosis with bulging visible at day 22 and complete transformation at day 30. **C**, Proportion of sporozoites versus EEF-like's of wild type, *puf1-* and *puf2-* parasites in mosquito SG's during 13 days of salivary gland infection. Scale bars = 10 µm.

Together these data suggested that lack of either Puf1 or Puf2, or both proteins has no, or at most very minor effects on the majority of the different life cycle stages of *P. berghei*, including the number of sporozoites reaching mosquito salivary glands. However, microscopic examination of salivary gland 30 days after mosquito infection revealed aberrant morphology of *puf2-* parasites (Fig. 2; Videos S1 [wild type], S2 [*puf1*-], S3 [*puf2-*]). Sporozoites of both independent *puf2-* mutants at day 22 after mosquito infection and later, began to round up and progressively resembled early hepatic stages (Fig. 2A, B; Fig. S6); by day 24 after mosquito infection the majority of parasites in mosquito salivary glands were morphologically similar to early EEF's (76.49±2.43%) (Fig. 2C) with an average bulging area of 4.72±0.42 µm^2^ that increased to 6.02±1.14 µm^2^ on day 30 of infection. The bulging area of older *puf2-* parasites is comparable in size to 8–10 hours liver stage EEF's. On the other hand *puf1*- and wild type salivary gland sporozoites (SGS) remained typically slender throughout the entire period (Fig. 2). *puf1*-/2- parasites recapitulated the *puf2-* single KO phenotype (Fig. S6).

### 18-day *puf2-* sporozoites are defective in motility, cell traversal and infection

**Figure 3:**
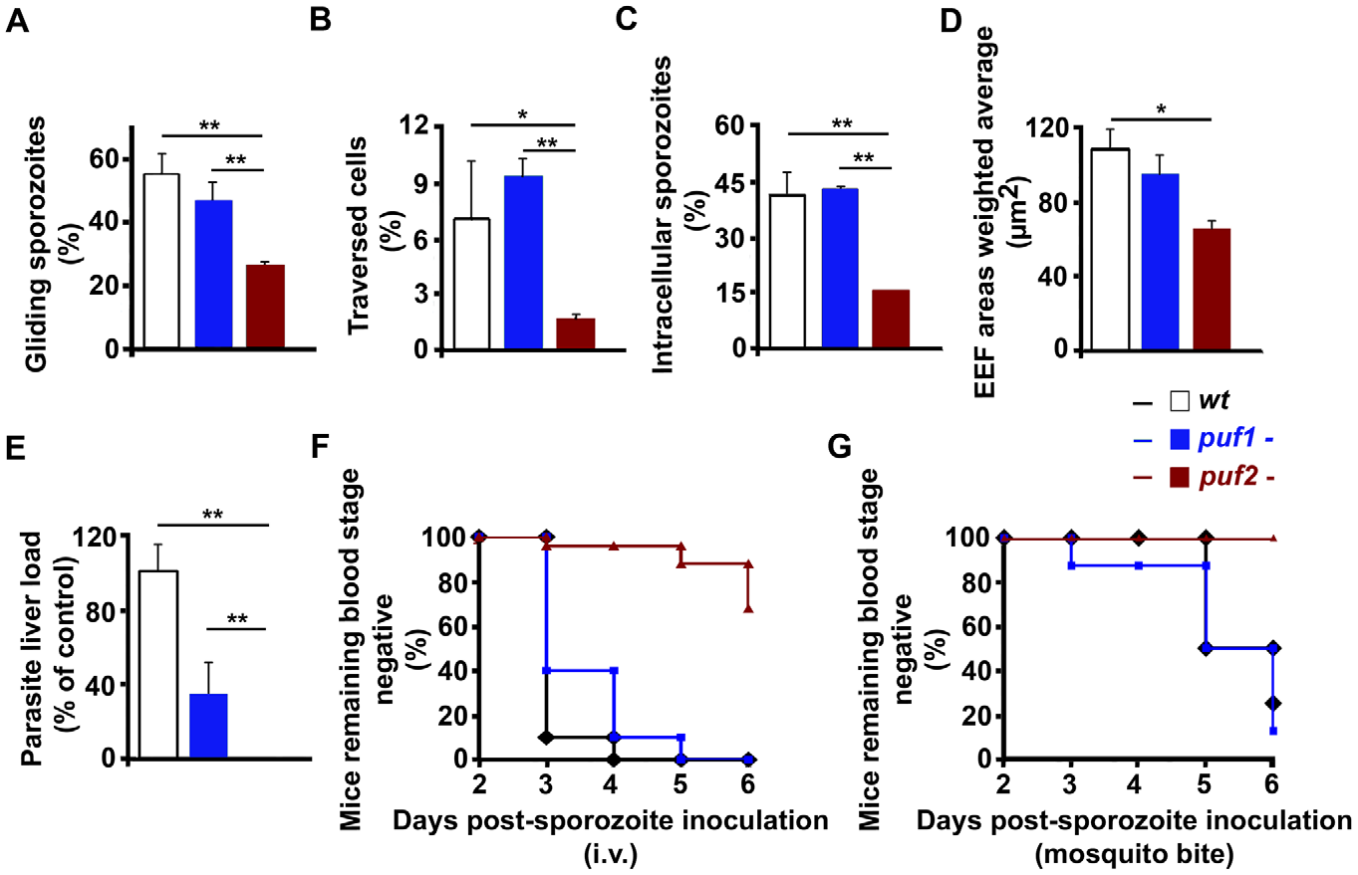
Functionality and liver stage infectivity of wild type and mutant sporozoites. **A**, 3×10^4^ sporozoites were allowed to glide on cover slips pre-coated with anti-circumsporozoite protein (CSP) antibody for 40 minutes at 37°C. The number of parasites associated with CSP trails is a measure of gliding motility ability. **B**, Dextran tetramethylrhodamine was added to Huh7 cells before sporozoite addition. Two hours p.i. the number of traversed cells (Dextran+) was quantified by fluorescence-activated cell sorting. **C**, Huh7 cells fixed and double-stained with anti-CSP antibody to distinguish extracellular from intracellular sporozoites after a 2-hour incubation with 3×10^4^ sporozoites. **D**, EEF development 48 h after addition of sporozoites to Huh7 cells. EEF areas were quantified with Image J of fluorescence microscopy images. For Figure 3A-D, n = 3. **E**, *Plasmodium* liver load 44 h after intravenous injection of 1×10^4^ sporozoites; parasite load measured by qRT-PCR of *P. berghei* 18S rRNA normalized to hypoxanthine-guanine phosphoribosyltransferase. Five C57Bl/6 mice per group. **F**, Appearance of blood parasitaemia following infection of C57Bl/6 with 1×10^4^ intravenously injected sporozoites (wild type n = 10 mice; *puf1-* n = 10; *puf2-* n = 25). **G**, Appearance of blood parasitaemia after mosquito bite (wild type n = 4 mice; *puf1-* n = 8; *puf2-* n = 10). All experiments used sporozoites 18 days after mosquito infection. T-test * p<0.05; ** p<0.01. All data show mean±SD.

At day 18 *puf2-* sporozoites had reached the mosquito salivary glands in similar numbers as *puf1*-, *puf1*-/2- and wild type parasites (Fig. S5) and did not present obvious morphological changes. Although superficially morphologically identical to wild type sporozoites, 18 day *puf2-* SGS displayed significantly reduced gliding motility and cell traversal ability when compared to *puf1*- and wild type parasites (Fig. 3A, B; t-test *p*<0.05). Consequently, *puf2-* SGS were less infective *in vitro* to Huh7 hepatoma cells (Fig. 3C; t-test *p*<0.05) and parasites that had successfully invaded, showed delayed development (Fig. 3D; t-test *p*<0.05). When we compared parasite loads in mouse livers infected 44 hours earlier after intra-venous injection of identical numbers (n = 10,000) of day 18 *puf1*-, *puf2-* or wild type sporozoites we found a significant impairment of liver infection by *puf2-* when either compared to WT or *puf1*- parasites (Fig. 3E; t-test *p*<0.05). Ten days after i.v. injection of SGS all mice infected with wild type and *puf1*- SGS developed blood stage parasitemia, while only 32% of the mice infected with *puf2-* parasites did (Fig. 3F). During infection by mosquito bite, blood stage parasites became patent only in mice infected with wild type and *puf1*-, but never with *puf2-* parasites (Fig. 3G). Although mice infected with *puf1*- SGS show a lower parasite liver load than mice infected with WT SGS (Fig. 3E), no differences were found in blood stage patency (Fig. 3F and G). Throughout, the behavior of the *puf1*-/2- parasite was similar to the *puf2-* parasite, which suggests that all defects are attributable to the lack of Puf2, with no additional effects arising from the simultaneous deletion of both genes (Table S2).

### Transcriptome changes precede visible morphological changes of *puf2-* sporozoites

**Figure 4:**
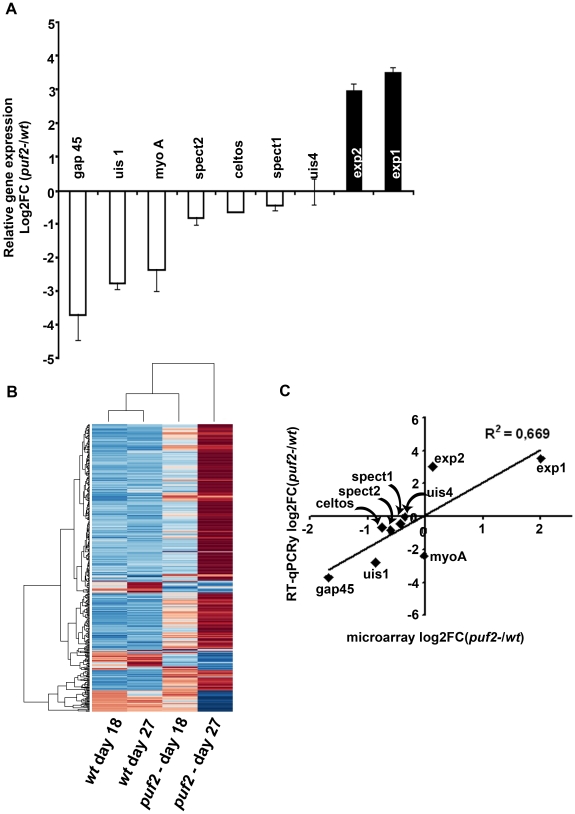
Transcriptional changes in *puf2-*. **A**, Quantitative RT-PCR analysis was done on cDNA from wild type and *puf2-* salivary gland sporozoites (SGS) at day 18 after mosquito infection. **B**, Heatmap of expression changes measured by microarray analysis for >300 genes in wild type and *puf2-* at days 18 and 27 after mosquito infection. Expression values are scaled up to the rows and range from -3 (blue) to +3 (red). **C**, Correlation analysis of quantitative RT-PCR and microarray results.

The phenotypic analyses of day 18 *puf2-* SGS suggested that premature de-differentiation of sporozoites could already have been initiated prior to the visible manifestation of the morphological changes evident in older parasites; we reasoned the absence of Puf2 might affect the steady state transcriptome; intra-hepatic and axenic differentiation of SGS into EEF's is correlated with distinct transcriptome adaptations [13], [14], [30]. Therefore we compared in 18-day SGS by RT-PCR (data not shown) and RT-qPCR the expression profiles of genes known to be transcribed in sporozoites, or in EEF's but not in sporozoites (e.g. *exp-2* - PBANKA_133430 and *exp-1 -*PBANKA_092670) in comparison to *ama-1* (PBANKA_091500). Transcripts of the sporozoite genes *gap45* (PBANKA_143760), *myo-a* (PBANKA_135570), *spect2* (PBANKA_100630), *celtos* (PBANKA_143230) and spect1 (PBANKA_135560) – their protein products are important for gliding motility and cell traversal [8] – were less abundant in *puf2-*; *uis-4* (PBANKA_050120) showed no marked difference (Fig. 4A). Conversely, the liver stage genes exp-1 and *exp-2* (a constituent of the PTEX translocon of exported PEXEL-containing proteins [31]) were clearly more abundant in the absence of Puf2. *uis-1/ik2* (PBANKA_020580), a kinase reported to regulate translational capacity of salivary gland sporozoites [32] was also down-regulated. Together these differences in mRNA indicated that the changes in morphology of the older *puf2-* salivary gland parasite are indeed preceded by changes in steady state mRNA levels in superficially normal, younger SGS. The down-regulation of *myo-a*, *gap45*, *celtos*, *spect1* and *spect2* could explain why day 18 *puf2-* sporozoites are deficient in gliding motility as well as infection (Fig. 3).

### *puf2-* parasites in salivary glands show progressive transcriptome changes

The RT-PCR data prompted the analysis of the global transcriptome variations in *puf2-* sporozoites. We compared mRNA levels obtained from both day 18 and day 27 wild type and *puf2-* parasites by microarray hybridization using 3 biological replicates from each time-point. In total, our analyses showed that in the absence of Puf2, 267 genes were up-regulated (UR) at either days 18 or 27 or both, while 47 were down-regulated (DR) at least 1.5-fold (F-test, *p*<0.05; Fig. 4B; Table S3A); these genes included those that we initially identified in the RT-qPCR survey (Fig. 4C; Fig. S7).

Our data indicate an overall increase in transcriptional activity in mutant parasites which is suggested by the larger number of UR versus DR genes (66 vs. 14 at day 18; 271 vs. 47 at day 27, on a pairwise basis, *p*<0.05). Still, the DR transcript data set contains genes that encode components of the inner membrane complex and enzymes of the TCA cycle (Fig. S8), as well as genes with a well-documented role in motility and invasion, reflecting the observed functional deficiencies (see Fig. 3) in the *puf2-* parasites. These genes include *celtos*, *spect1*, *spect2*, *tlp1 (PBANKA_111600)*, *trsp (PBANKA_020910)*, *siap1 (PBANKA_100620)*, *mtrap (PBANKA_051280)*, *trep (PBANKA_130650), psop9/gama (PBANKA_070190), gap45, and p36p* (PBANKA_100220; Table S3B); another 10 conserved, but uncharacterized *Plasmodium* proteins that contain a signal peptide, trans-membrane domain(s) or GPI anchor are also DR, maybe indicating a function during the hepatocyte invasion process.

On the other hand, UR genes fit in the categories of DNA metabolic processes, ribonucleoprotein complex, ribosome/translation and protein folding (Fig. S8). Of the 7 differentially expressed transcription factors found 6 are UR and include TFIIH (PBANKA_141340), the RNA polymerase II subunit (PBANKA_020330), 2 putative transcription factors (AP2's, PBANKA_083520 and PBANKA_010950), and 2 TFIIS Zinc-fingers (PBANKA_030420 and PBANKA_142110; Table S2C); concomitantly mRNA capping and splicing factors, and genes involved in ribosomal and transfer RNA processing (n = 16) are almost exclusively UR (Table S2D). Throughout, translation factors and ribosomal proteins (n = 52; Table S3E) are UR in *puf2-*, while *ik2* (a negative regulator of translation through phosphorylation of eIF2α) is DR, consistent with the observed increase in protein translation in IK2 null mutant sporozoites [32]. In parallel, many chaperones (n = 17; Table S3F) and genes with protein transport-related functions (n = 29; Table S3G) are UR; these include for example *plasmepsin V* (PBANKA_133870) – the PEXEL-motif cleaving enzyme– and *exp2* [31], [33]. 14 genes linked to the ubiquitin-proteasome system are UR at day 27 in *puf2-* parasites (Table S3H) which supports an involvement in the observed elimination of rhoptries and micronemes during metamorphosis [34]. Additional UR genes in the *puf2-* mutant include mitochondrial and fatty acid synthesis genes (Table S3I and J). Finally we observe an increase in replication factors, *rad51 (PBANKA_093950)*, histone *h2b* (PBANKA_094180) and *alba-3* (PBANKA_120440; n = 17, Table S3K).

In summary, the microarray analysis emphasizes genes involved in increased metabolic activity to be UR in EEF-like mutant parasites. The comparison between wild type SGS from days 18 and 27 post-mosquito infection on the other hand showed almost no transcriptome alterations. Only 6 and 16 genes, respectively, were UR or DR out of the total of approximately 5400 *P. berghei* genes; none of them however significantly (moderated t-test *p*>0.05; Table S3A). This clearly suggests that the wild type parasites' quiescent yet infective status with respect to transcription and mRNA abundance is maintained for at least 10 days while residing in the mosquito salivary gland. A comparison with the sporozoite and EEF proteomes of the related, rodent malaria species *P. yoelii* [35] showed that 89.7% (96/107) of UR *P. berghei* mRNAs in *puf2-* sporozoites (at day 18 and 27) are indeed detected only in *P. yoelii* liver stage parasites but not in SGS, corroborating the notion that *puf2-* sporozoites in fact resemble genuine, early liver stage parasites (Table S3A).

### *puf2-* parasites in salivary glands exhibit ultrastructural features of early stage EEFs

**Figure 5:**
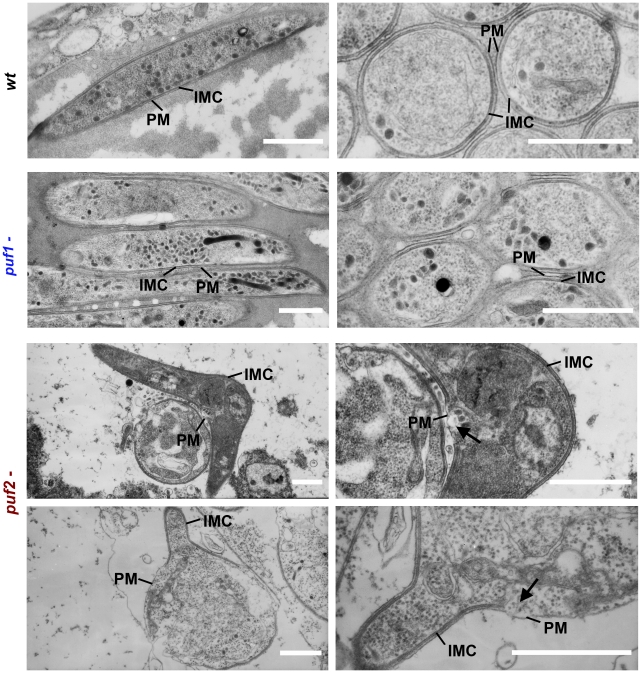
Ultrastructural evidence of *puf2-* parasites transforming into early liver stage EEFs in *A. stephensi* mosquito salivary glands. Transmission electron microsocopy of 29-day salivary gland parasites showing the presence of slender-shaped, wild type and *puf1-* sporozoites with outer plasma membrane (PM) and entirely intact, inner membrane complex (IMC). *puf2-* salivary gland parasites developing into early EEFs show clear IMC disruption in the bulging region (arrows). Left pictures: longitudinal sections; right picture: transversal sections. Scale bars = 1 µm.

Further evidence of the genuine nature of older *puf2-* parasites as early liver stage parasites arose from ultrastructural studies. The bulge formation in the center of parasite is a hallmark of the initial differentiation process during hepatic development. Importantly, it has been shown to coincide with a loss of the inner membrane complex (IMC) associated with motility and rounding-up [34]. When we analyzed wild type and *puf2- P. berghei* by EM we found a clear sign of IMC disruption in day 27 *puf2-* parasites (Fig. 5) but not in day 18 *puf2-* SGS (Fig. S9). Neither wild type nor *puf1*- showed any signs of IMC impairment (Fig. 5). The resulting protrusions mark the beginning of sporozoite differentiation into liver stage trophozoites and occur approximately 4 hours after liver cell infection, following degradation of IMC components [34] such as Alveolin 9 (PBANKA_124060) or MyoA. All these changes in *puf2-* SGS further confirm the profound developmental switch in response to the lack of Puf2.

### *Plasmodium* sporozoite transformation into EEFs is protein translation dependent

Overall, our data show that Puf2 is a master regulator of *Plasmodium* developmental control during transmission from the mosquito vector to the mammalian host. The highly conserved nature of the Pumilio homology domain (PHD) of the *Plasmodium* proteins [27] (Fig. S1), its conserved function in many different organisms [5], [22], [36], [37], [38], the capacity of *P. falciparum* Puf2 to bind RNA *in vitro* [26] and its localization to few cytoplasmic speckles in *P. berghei* sporozoites (Fig. 1B) strongly suggested that sporozoite latency in *Anopheles* salivary glands relies on the control of protein translation through a post-transcriptional mechanism.

**Figure 6:**
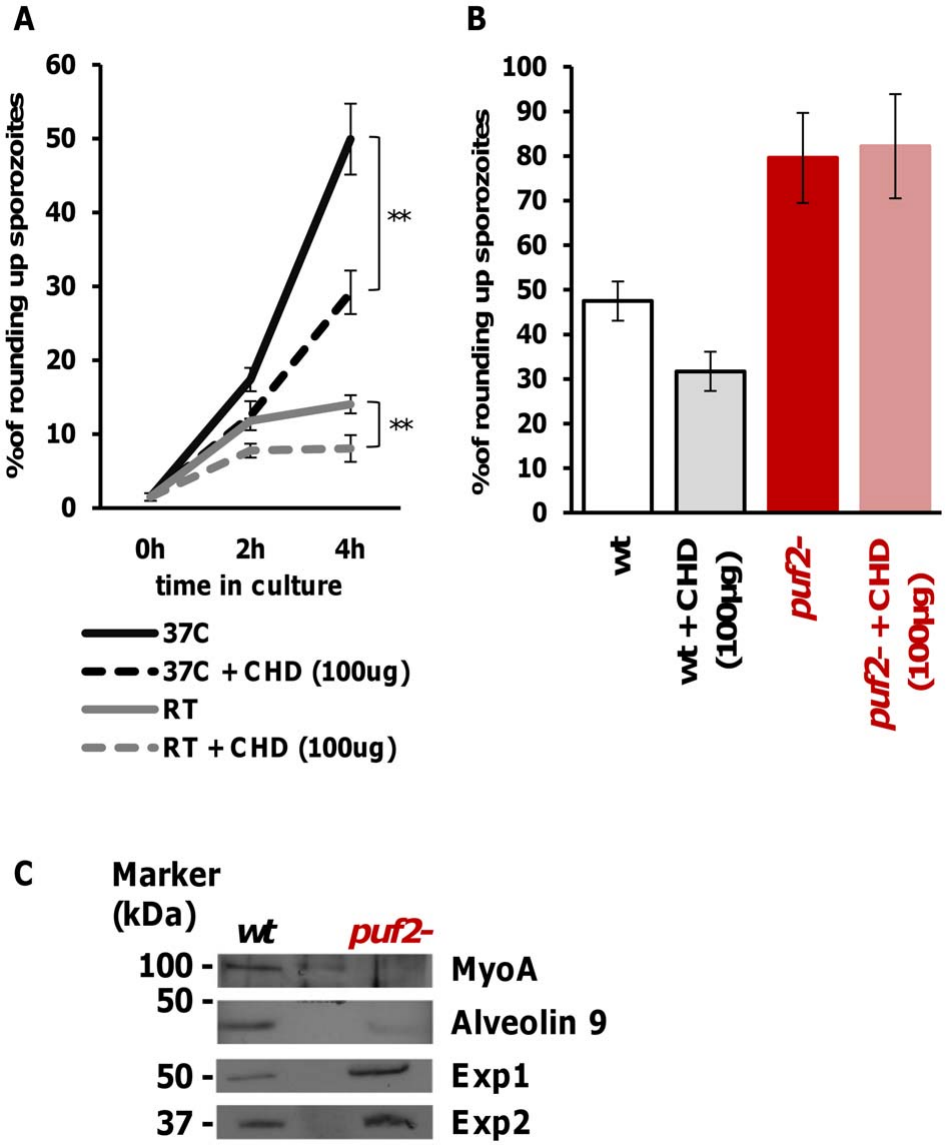
*Plasmodium* sporozoite transformation into EEF-like form is protein translation dependent. **A,** Wild type 18 day SGS were incubated at 37°C or room temperature (RT) for 2 h and 4 h and with or without the presence of cycloheximide (CHD, 100 µg/ml) as indicated. Transformation of WT SGS into EEF-like *in vitro* is more pronounced at 37°C for 4 h and relies on protein translation. T-test ** p<0.01. All data show mean±SD. **B,** WT and *puf2-* 18 day SGS were incubated at 37°C for 4 h with and without the presence of cycloheximide (CHD, 100 µg/ml) as indicated, showing that transformation of *puf2-* sporozoites does not rely on protein translation. **C,** Protein expression in *puf2-* and wild type SGS, 18 days after infection.

The notion that SGS to EEF transformation is dependent on *de novo* protein synthesis was supported by the ability of cycloheximide (a general protein synthesis inhibitor) to significantly reduce the metamorphosis of wild type SGS into EEF-like parasites (Fig. 6A) in an established *in vitro* transformation assay [15]. In this assay, sporozoites transform into EEF-like parasites within 1–2 h when placed at a temperature of 37°C (Fig. 6A). Importantly, comparison of transformation of WT and puf-2 sporozoites showed that puf-2 parasites produced almost twice as many early EEF's compared to WT after 4 h incubation at 37°C in a cycloheximide-insensitive manner (Fig. 6B). This result implies that, in *puf*2- parasites, proteins required for SGS transformation into EEF-like parasites have already been produced by day 18 of mosquito infection. Indeed, Western blots performed with 18 day wild type and mutant SGS showed that transformation-associated changes in protein level had already occurred (Fig. 6C): proteins involved in sporozoite motility (MyoA) or IMC maintenance (Alveolin 9) are clearly less abundant in mutant SGS whilst proteins typical of EEF development (Exp1 and Exp2) are readily detected prior to any morphological changes.

Our data clearly show that *Plasmodium* sporozoite transformation into EEFs is protein synthesis dependent, and sporozoite quiescence relies on post-transcriptional control and the RNA binding protein Puf2.

## Discussion

Following the mosquito blood meal, sporozoites that manage to invade a host hepatocyte quickly initiate differentiation into liver stage trophozoites; these early changes are characterized by a de-differentiation process that involves loss of the inner membrane complex, protrusions in the region of the parasites nucleus and loss of internal organelles [34]. A single parasite ultimately multiplies by a factor of a few 1000-fold within 48 hours in *P. berghei* to give rise to first generation merozoites. *Plasmodium* development in the liver is accompanied by clear host cell transcriptional changes and adaptations that reflect the parasite's needs [39]; these include generation and maintenance of the parasitophorous vacuole, the export of proteins such as circumsporozoite protein (CS) into the host cytoplasm [40] and possibly uptake of exogenous lipids [41], replication and differentiation to form merozoites.

On the other hand, the transcription of *Plasmodium* genes essential for full intra-hepatic development is triggered by a temperature shift and contact with host cells [13], [14]. Transformation into early EEF's can to some degree be recapitulated in the absence of host cells; for this, wild type SGS require merely the presence of serum or bicarbonate and a temperature shift from the mosquito's body temperature (≈21°C) to the mammalian host's 37°C [13], [15]. However, our data clearly show that in the absence of the RNA binding protein Puf2, initiation of sporozoite to EEF metamorphosis takes place inside mosquito salivary glands without the need for environmental cues received during transmission from the mosquito vector to the mammalian host. Although the morphological changes observed at the light-microscope level could be interpreted as resulting from non-specific degenerative processes, our data on specific transcriptional changes, changes in protein synthesis and ultrastructural features indicate that the phenotype of *puf2-* SGS is the result of a specific differentiation process into EEFs. Indeed, the alterations in transcription, protein expression and ultrastructural features in *puf*2- sporozoites match those occurring in early liver stage forms [30], [42] and are not easily reconciled with random degenerative processes. Importantly, the cellular and molecular events leading to the metamorphosis of *puf*2- SGS into early hepatic stages occur prior to any apparent morphological changes, as shown by the loss of infectivity of *puf2-* SGS before any morphological changes are manifest.

In many organisms, Puf proteins inhibit translation of specifically recognized mRNAs (generally a small number), either by repressing their translation or enhancing decay [1]. Consistent with this conserved biological function, we show that *Plasmodium* Puf2 is localized to few cytoplasmic speckles and possesses a highly conserved Pumilio homology domain (PHD). Together with our transcriptional analyses, these data strongly suggest that Puf2 is regulating the translational efficiency of one or several unknown key factor(s); we hypothesize that once translationally activated such proteins quickly direct the developmental progression from SGS to early hepatic stages.

**Figure 7:**
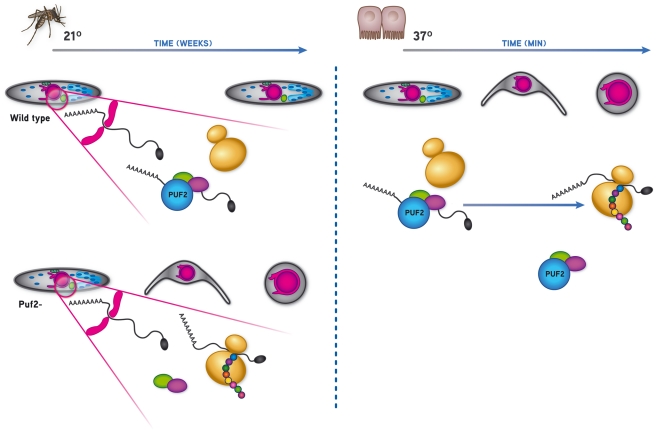
Proposed model for Puf2-mediated regulation of *Plasmodium* developmental progression during transmission from the mosquito vector to the mammalian host. In the mosquito Puf2 controls the translation of key protein(s). In its absence, this(ese) proteins are translated and sporozoites initiate transformation into the round EEF, which only occurs in the mammalian host in wild-type parasites.

Recently, the sporozoite's latency status was reported to rely on mechanisms akin to mammalian and yeast stress granule formation with a phosphorylation dependent inhibition of protein translation [32]. Absence of eIF2α phosphorylation in a mutant lacking expression of the PBIK2 protein–the *pbik2* gene was originally identified as *upregulated in sporozoites 1* or *uis1* [43]–was shown to result in an approximately 2-fold increase in translation as measured by ^35^S-Met/Cys incorporation in sporozoites at 25°C, and 3-fold at 37°C. However, close observation of *ik2-* parasites in *Anopheles* salivary glands revealed that only 11.6±8% parasites show signs of transformation by day 30 of infection, while more than 99% of *puf2-* parasites are already fully rounded up by that time (Fig. S10). Thus, despite a significant increase in protein translation in the absence of PBIK2, *pbik2-* sporozoites do not initiate the program of transformation as significantly as *puf2-* sporozoites. This difference in phenotype could be explained by a dominant role of Puf2 in binding to essential mRNAs repressing their translation into proteins that are needed for the transformation program to occur; in our proposed model (Fig. 7), we speculate the absence of Puf2 is consistent with the translation of these essential transcripts thereby triggering premature metamorphosis. This may alleviate the translational repression promoted by IK2 [32], perhaps involving protein phosphatase 2C (PBANKA_091340) [44] which is strongly up-regulated in *puf2-* parasites at day 18 of mosquito infection (Table S3L). Although we have very limited data on proteome changes in the *puf2-* parasites, our Western analyses indicate that changes (both up and down) in steady state protein levels do occur. It remains unclear whether Puf2 independent translation is mediated through eIF2α, although *ik2* is already significantly decreased in *puf2-* parasites and protein phosphatase activity might be increased by day 18 of infection.

At present the nature of the mRNAs directly regulated by Puf2 are unknown; an exploratory MEME analysis of DR transcripts identified at all time points was inconclusive, most likely due to the fact that both the consensus Pumilio recognition motif (UGUAAA/UAU) and untranslated regions of *P. berghei* mRNAs are extremely AU-rich; out of the 374 transcripts detected to be significantly de-regulated in the *puf2* gene deletion mutant, 106 have at least 1 NRE (Nanos Response Element) within 400 nucleotides of the stop codon. Statistically, there is no enrichment for NRE's in up or down-regulated genes (chi-square, df = 1, p = 0.2238; Table S3N) with the important caveat that actual 3′ UTRs have rarely been mapped in *P. berghei*; hence these bio-informatic results are very speculative and identified NRE's may not exist in mature mRNAs. Nonetheless, Puf2 clearly maintains SGS on a “stand-by”, quiescent mode until they have invaded mammalian host hepatocytes where they expand into first-generation, blood-infectious merozoites. Thus, Puf2 constitutes a key player in the developmental control during a critical time-point of the *Plasmodium* life cycle, the malaria parasites' transmission from the invertebrate to the vertebrate host. Altogether, and considering the highly conserved nature of PUFs, this shows that post-transcriptional events are central to the major developmental switches that are associated with host transition during the *Plasmodium* life cycle.

## Materials and Methods

### Laboratory animals

This study was carried out in strict accordance with the recommendations of both the Animal Experiment Committees governed by section 18 of the Experiments on Animals Act and registered by the Dutch Inspectorate for Health, Protection and Veterinary Public Health (Ministry of Health, Welfare and Sport), and the Portuguese official Veterinary Directorate, which complies with the Portuguese Law (Portaria 1005/92). The Dutch and Portuguese Experiments on Animal Act strictly comply with the European Guideline 86/609/EEC and follow the FELASA (Federation of European Laboratory Animal Science Associations) guidelines and recommendations concerning laboratory animal welfare. In The Netherlands, all animal experiments were approved by the Animal Experiments Committee of the LUMC (ADEC). In Portugal, all animal experiments were approved by the Portuguese official veterinary department for welfare licensing and the IMM Animal Ethics Committee. All experiments were carried out using Swiss-OF1 female mice (OF1-ico, Construct 242; age 6 weeks old, Charles River Laboratories International, Inc), C57Bl/6 and BALB/c mice (6–8 weeks of age; Harlan Laboratories, Inc. or Charles River Laboratories International, Inc). All efforts were made to ensure minimal suffering to the animals.

### Generation of *puf1* (gene model PBANKA_123350), *puf2* (gene model PBANKA_071920) and *puf1*/*puf2 P. berghei* gene deletion mutants

*puf1* and *puf2* were targeted for disruption by standard double-crossover homologous recombination with linearized targeting plasmids. Transfection and drug selection of mutant parasites was performed using standard technology of genetic modification developed for *P. berghei* [45], [46]. Cloned parasite lines were obtained by limiting dilution. Plasmid integration into the genome was verified by Southern analysis of separated chromosomes and diagnostic PCR; the absence of transcript was confirmed by Northern analysis. For details of vectors, targeting regions, and primers used see Figs. S2 and S3, as well as Tables S4-7. Plasmids pL0001 and pL0035 can be obtained from http://www.mr4.org. Details for all Rodent Malaria genetically modified *P. berghei* lines used in this study can be found in the RMgm database (http://www.pberghei.eu).

For *puf1* gene deletion, PCR-amplified 5′ and 3′ targeting regions were cloned into plasmid pL0001 yielding pAB60 (containing the pyrimethamine *tgdhfr/ts* selection marker), or plasmid pL0035 yielding pL1214 (containing the pyrimethamine/5-fluorocytosine *hdhr/yfcu* positive/negative selection marker). Mutant 351cl1 (pAB60; *puf1-a*; RMgm-513) was generated in the GFP-reference line cl15cy1 [45], mutant 900m2cl3 (pL1214; *puf1-b*; RMgm-514) was generated in the GFP+ reference line 507cl1 (RMgm-7 at http://www.pberghei.eu).

The selection cassette (*hdhfr*/*yfcu*) in 900m2cl3 was removed by negative selection [47]; four mice infected with parent population 900 were treated with 5-fluorocytosine (5-FC) at a parasitemia of 0.1–0.5% with a single, daily 20 mg/ml dose (0.5 ml) for a period of 4 days. Resistant parasites were collected at days 5–7 and analyzed by diagnostic Southern analysis to confirm removal of the drug-selectable marker *hdhfr/yfcu* by a recombination event between the two 3′*-dhfr-ts* sequences (Fig. S2). A PCR amplified fragment of the 3′*-dhfr-ts* region was used for Southern analysis (the primer sequences are provided in Table S5). Parasites from mouse 2 were cloned by limiting dilution, resulting in mutant 900m2cl3 (*puf1-b*).

For *puf2* gene deletion, PCR-amplified 5′ and 3′ targeting regions were cloned into plasmid pL0001 yielding pAB70 (containing the pyrimethamine *tgdhfr/ts* selection marker), or plasmid pL0006 yielding pL1317 (containing the pyrimethamine *hdhfr* selection marker). Mutant 375cl1 (pAB70; *puf2-a*; RMgm-515) was generated in the GFP-reference line cl15cy1 [45], mutant 1267cl2 (pL1317; *puf2-b*; RMgm-516) was generated in the GFP+ reference line 507cl1 (RMgm-7 at http://www.pberghei.eu).

The following probes were used for Southern analysis: PCR-amplified fragments for the *hdhfr* and *tgdhfr-ts* genes (for primer sequences see Table S7) and a *puf2* sequence consisting of the 0.4 kb EcoRI/HincII *puf2* fragment; in *puf2-* this part is deleted.

In experiment 1081 we generated a mutant line in which both *puf1* and *puf2* were deleted. To generate mutant 1081cl1 (*puf1-/2-*; RMgm-591) the selectable marker cassette *hdhfr-yfcu* was first removed from mutant 900 (*puf1-b*) by negative selection essentially as described [47]. In brief, 4 mice infected with mutant 900 were treated with 5-fluorocytosine (5-FC) starting at a parasitemia of 0.1–0.5% with a daily single dose of 0.5 ml of a solution of 20 mg/ml day for a period of 4 days. Resistant parasites were collected between days 5–7 after start of the 5-FC treatment and the genotype analyzed by diagnostic Southern analysis to confirm removal of the drug-selectable marker *hdhfr-yfcu* by a recombination event between the two 3′ *pbdhfr/ts* sequences (Fig. S2). Parasites from one of the four mice (mouse 2) that had been treated with 5-FC were cloned by limiting dilution, resulting in mutant 900m2cl3 (*puf1-b*). Parasites of line 900m2cl3 were then transfected with vector pL1317 for disruption of *puf2* (Fig. S3). Selection and cloning of transformed parasites resulted in mutant 1081cl1 (*puf1-/2-*) in which both *pumilio* genes are disrupted.

### Gene expression analysis by Northern blot and RT-PCR

Total RNA was isolated from blood stage parasites from asynchronous and synchronized infections [48] and analyzed by Northern hybridization. Northern blots were hybridised with *puf1* and *puf2* PCR-amplified fragments (for primer sequences see Tables S5 and S7). As loading control, blots were hybridized with *p28* (PBANKA_051490) or with primer L644R specific for the blood stage, large subunit ribosomal RNA [49].

For RT-PCR, total RNA was isolated from highly purified gametocytes and day 18 and 27 sporozoites and reverse transcribed with hexamers and oligo d(T) oligonucleotides; primers were 479 and 480 for *puf1*, and 477 and 478 for *puf2*, in both cases spanning an intron (Table S8).

### Asexual growth rate, gametocytogenesis and gametogenesis

The *in vivo* multiplication rate of asexual blood stage parasites was determined during the cloning procedure and calculated as follows: the percentage of infected erythrocytes in Swiss OF1 mice injected with a single parasite is determined at day 8 to 11 by counting Giemsa stained blood films; the mean asexual multiplication rate per 24 h is then calculated assuming a total of 1.2×10^10^ erythrocytes per mouse (2 ml of blood). The percentage of infected erythrocytes in mice infected with wild type reference lines of the *P. berghei* ANKA strain typically ranges between 0.5–2% at day 8 after infection, resulting in a mean multiplication rate of 10 per 24 h [50], [51].

Gametocyte and gamete production were determined following standardized conditions [48]. Gametocyte production is defined as the Gametocyte Conversion Rate which is the percentage of ring forms that develop into mature gametocytes in synchronized infections in mice treated with phenylhydrazine. Male gamete formation is defined as the percentage of male gametocytes that form gametes after *in vitro* induction by exflagellation; exflagellating male gametocytes are counted in a Bürker cell counter 15 to 20 minutes after induction. Female gamete formation is defined as the percentage of female gametocytes that emerge from the red blood host cells after *in vitro* induction of gametogenesis; free female gametes were counted in Giemsa stained smears made 20 minutes after induction. The fertility of wild type and mutant gamete populations was analysed by standard *in vitro* fertilisation and ookinete maturation assays [52], [53] from highly pure gametocyte populations [54]; the fertilisation rate of gametes is defined as the percentage of female gametes that develop into mature ookinetes determined by counting female gametes and mature ookinetes in Giemsa stained blood smears 16–18 h after *in vitro* induction.

Human hepatoma cell line Huh7. Huh7 cells were cultured in RPMI medium supplemented with 10% fetal calf serum (FCS), 1% non-essential amino acids, 1% penicillin/streptomycin, 1% glutamine and 10 mM Hepes, pH 7 and maintained at 37°C with 5% CO2. All consumables were obtained from Gibco/Invitrogen.

### *Anopheles stephensi* mosquito maintenance

*A. stephensi* were bred at the insectary of the Instituto de Medicina Molecular (IMM). All life cycle associated experiments (mosquito infection, *in vitro* Huh7 infection, *in vivo* mouse infection) presented in this paper were performed with GFP+ *puf1*- clone 900m2cl3 and *puf2*- clone 1267cl2 and confirmed with GFP- *puf1-* 351cl1 and *puf2-* 375cl1.

### *Anopheles stephensi* mosquito infection and analysis of parasite development

1×10^6^ infected red blood cells of *P. berghei* wild type (259cl2; RMgm-5; GFP+) [52] and mutant lines, *puf1-*and *puf2-* were intraperitoneally injected in BALB/C mice . Four to 5 days later, when at least one exflagellation event was observed per microscope field, mosquitoes were allowed to feed on anaesthetized mice for 0.5–1 h on two consecutive days. At day 10 post blood meal, 9 infected midguts were removed and the number of oocysts per midgut determined by fluorescence microscopy. Parasites per salivary gland (SG) were quantified in 3 independent transmission experiments in which 9 infected mosquitoes per experiment, from 19 to 22 days after mosquito infection for each genotype, were dissected; three groups of 3 SGs for each experiment for each genotype were smashed and the number of parasites per SG quantified in a Neubauer chamber.

### Quantification and morphological analyses of mutant sporozoites

Three A. *stephensi* SGs infected with *wild type*, *puf1*- or *puf2*- parasites were removed on days 18, 22, 24, 27 and 30 after infection. SGs were smashed to release the parasites, and the proportion of sporozoites to EEFs-like quantified. On day 30 of mosquito infection, whole infected SGs were mounted in glass bottom culture dishes (MatTek Corporation); a Zeiss LSM 510 META confocal microscope (Zeiss, Oberkochen, Germany) was used to perform a Z-series scan followed by 3D reconstructions of the infected SGs using Imaris (Bitplane AG, Switzerland).

### *In vitro* sporozoite hepatocyte infectivity

For all experiments, wild type, *puf1-* and *puf2-* salivary gland sporozoites were collected on day 18 after the mosquito blood meal.

Sporozoite gliding was evaluated with 3×10^4^ sporozoites for 40 minutes in complete RPMI, at 37°C on glass cover slips covered with anti-circumsporozoite protein (CSP) monoclonal antibody [3D11; 53]. Sporozoites were subsequently fixed in 4% paraformaldehyde (PFA) for 10 minutes and stained with anti-CSP. The percentage of sporozoites associated with CSP trails was quantified by fluorescence microscopy.

Cell traversal assays were performed with 3×10^4^ sporozoites added to 7×10^4^ Huh7 cells (seeded on the previous day) in the presence of 1 mg/ml of cell-impermeable dextran tetramethylrhodamine (10 000 MW), lysine fixable (fluoro-ruby) (Molecular Probes/Invitrogen). After 2 hours, the percentage of dextran-positive cells was quantified by fluorescence-activated cell sorting (FACS)[55].

In order to quantify cell invasion, 3×10^4^ sporozoites were added to Huh7 cells. Infection was stopped after 2 h by addition of PFA 4%; double staining with anti-CSP was performed according to [56] in order to distinguish extracellular from intracellular sporozoites. Intra-hepatic development was assessed by fixing infected Huh7 cells at 48 h p.i. with 4% PFA. Parasites were stained with anti-GFP antibody conjugated with FITC (Molecular Probes/Invitrogen). Pictures were taken on an Axiovert 200 M fluorescence microscope and EEF size measured using ImageJ 1.38 h software.

### *In vivo* sporozoite infectivity

Male C57BL/6 mice (6–8 weeks) were intravenously (i.v.) injected with 1×10^4^ 18-day SG sporozoites (wild type, *puf1-* or *puf2-*). After 44 hours liver infection load was quantified by qRT-PCR analysis of *P. berghei* 18S rRNA normalized against hypoxanthine-guanine phosphoribosyltransferase (HPRT) (for primers see Table S4).

To assess mutant parasites capacity to pass through the liver and reach the blood, 1×10^4^ sporozoites were injected i.v. into C57BL/6 mice.

To verify mutant sporozoites infectivity during natural infection, C57BL/6 mice were exposed to 4 infected mosquitoes for 30 minutes. All mice were bitten by at least by one infected mosquito. Parasitemia were checked by Giemsa-stained blood smear daily until 10 days post infection.

### Electron microscopy

*A. stephensi* salivary glands infected with wild type, *puf1*- or *puf2*- parasites were removed and fixed in 2.5% gluteraldehyde in 0.1 M sodium cacodylate (pH = 7.3) for 48 hours at 4°C, followed by 3 10-minute washes in 0.1 M sodium cacodylate. All tissues were post fixed in 1% OsO_4_ in deionized water, washed and counterstained with uranil acetate for 30 minutes. After washing with de-ionized water for 10 minutes, dehydration with ethanol (70% and 96%, 1 minute each) was performed followed by 2 10-minute incubations in absolute ethanol and propylene oxide. Salivary glands were finally infiltrated with 1∶1 propylene oxyde and EPON resin for 30 minutes followed by overnight infiltration in 100% EPON's resin. The tissues were embedded in flat molds in 100 EPON for 48 hours at 70°C. Ultra-thin sections of 70 nm were cut with a diamond knife (Diatome 45°) in a ultra-microtome (Reichert Jung Ultracut-E), collected on copper grids (mesh 200 hexagonal) and stained with Reynolds lead citrate and 2% uranil acetate (5+5 minutes). The grids were observed on a Jeol JEM-100cxI transmission electron microscope.

### Expression profiling Reverse Transcriptase (RT)-PCRs and RT-qPCR

Wild type, *puf1-* and *puf2-* sporozoites were extracted at days 18 and 27 post mosquito infection; total RNA was extracted with TRIzol, and 400 ng total RNA reverse transcribed in the presence of random hexamers and oligo d(T) oligonucleotides with Superscript II. 25 ng were used in a PCR using the following cycling parameters: 94°C 3 minutes, 35 cycles of 94°C 10 seconds and 1 minute at 60°C, with a final elongation step of 10 minutes. PCR amplicons were run on 2% agarose gels. Oligonucleotide primers are shown in Table S3. Negative controls were performed with RT-negative samples (data not shown). RT-qPCR analyses were performed on cDNA prepared from day 18 wild type and *puf2-* salivary gland sporozoites; oligonucleotide primers are shown in Table S8. qPCR was done with Power SYBR Green (Applied Biosystems) according to the manufacturer's instructions. Three independent biological replicate cDNA samples were tested for each parasite. ABI 7500 Fast Sequence Detection System. Cycling parameters for all genes were: 95°C for 15 minutes, followed by 50 cycles of 95°C|15 seconds, 55°C|15 seconds, 60°C|45 seconds, followed by melting curve analyses. Relative mRNA abundance for each transcript was determined by the 2^−ΔΔCt^ method following ABI User Bulletin 2; expression data was normalised versus *ama-1*. Final values were log2 transformed to be comparable to subsequent microarray data.

### Expression profiling by microarray hybridization

The RMSANGER Affymetrix custom tiling array was designed against the 8 x genome assemblies for *P. berghei* and *P. chabaudi*. Prior to analysis, all 6.3 million probes were remapped using the exonerate software (http://http://www.ebi.ac.uk/~guy/exonerate) against the latest *P. berghei* genome assembly available from the Wellcome Trust Sanger institute (ftp://ftp.sanger.ac.uk/pub/pathogens/P_berghei/February_2011); all non-exact matches and redundant probes were discarded. A custom CDF file was generated using a combination of Perl scripts to analyse gene expression profiles of all ≈5000 annotated genes. 18 and 27 days sporozoites were dissected from salivary glands of *Anopheles stephensi* mosquitoes infected with wild type (ANKA GFPcon 259cl2) or *puf2-* (1267cl2 ). RNA from 3 independent infections each was extracted with TRIzol according to the manufacturer's instructions. Double amplified cDNA was synthesized using the Ambion WT Expression kit starting with ≈400 ng of mRNA and labelled using the Affymetrix Genechip WT Terminal Labeling and Hybridisation Kit according to the manufacturers' protocols. 18 hours hybridisations, washing, and staining were done according to Affymetrix recommendations. Genechip arrays were scanned with an Affymetrix 7G scanner. Raw scanned images were acquired using Affymetrix software suite GCOS and raw CEL files transferred to R/Bioconductor for pre-processing. The 3×wild type 18 days pi, 3×wild type 27 days pi, 3×*puf2-* 18 days pi and 2×*puf2-* 27 days p.i. hybridised arrays were background subtracted, quantile normalised and median polished using RMA [57]. An overall F-test was used to select for 374 variant genes using an adjusted p-value <0.05 (after correction for false discovery rate using the Bonferroni-Hochberg adjustment). A linear modelling was used to extract differential expression (DE) for each pair wise comparison using the Limma package [58]. Gene Ontology enrichment was tested using GOstats [59], GO.db (M. Carlson, S. Falcon, H. Pages and N. Li. GO.db: A set of annotation maps describing the entire Gene Ontology. R package version 2.3.5.) and GohyperGall function as described in [60] using the GO terms annotated for *P. falciparum* orthologs (version 5/31/2010, downloaded from http://www.geneontology.org/GO.downloads.annotations.shtml). All microarray gene expression data are presented in Table S3. Microarray gene expression for selected genes was validated with RT-qPCR (Fig. S7). Microarray data have been submitted to ArrayExpress under the accession number E-TABM-1067.

### Protein expression profiling by Western Blot

Wild type and *puf2-* sporozoites were extracted from mosquito salivary glands at day 18 post infection. An amount of protein corresponding to 300 000 sporozoites was loaded in each well of a 10% polyacrylamide gel and transferred to nitrocellulose membrane (Protran) by electroblotting. Protein expression levels were determined by incubating the membranes overnight at 4°C, with the following primary antibodies: anti-Exp1 (kindly provided by Volker Heussler), 1∶1000; anti-Exp2 (kindly provided by Paul Gilson and Brendan Crabb) 1∶1000; anti-Myo-A (kindly provided by Julian Rayner), 1∶300 and anti-Alveolin-9, 1∶300 and subsequent incubation with horseradish-peroxidase conjugated secondary antibody. Immunostained proteins were visualized with chemiluminescence detection (Thermo Scientific).

### Immunofluorescence assay of Puf2

Red fluorescent protein (RFP)+ sporozoites from the wild type reference line 733cl1 (RMgm-86) were dissected at day 23 post infection and washed once in 1X PBS (9300 rcf, 7 minutes, 4°C). 6500 parasites in 10 µl were allowed to adhere to polylysine slides, fixed for 15 minutes with 4% PFA, and washed 3×5 minutes with 1X PBS. After a 10-minutes wash with fresh 0.1 M Glycine buffer, sporozoites were permeabilized with 0.1% Triton-X100 for 10 minutes followed by a 3x5 minutes wash with 1X PBS. Slides were blocked 20 minutes at RT in 1% Albumin and incubated O/N with polyclonal rabbit anti-Puf antiserum (dilution 1∶300) upside down. Sera 904 and 905 were raised in rabbits immunised against FKDNLYNLKELNSW and ENLDKLKEETYILR at Eurogentec. Slides were washed 3x 15minutes in 1X PBS and incubated with donkey anti-rabbit, Alexa 488-conjugated secondary antibody, 30 minutes, 37°C (1∶400) again upside down. Slides were washed 3x 15 minutes in PBS 1X, and then incubated 3 minutes with DAPI, RT. Prior to mounting, slides were washed for 5 minutes and analysed with a widefield Zeiss Axiovert 200M microscope, with 63x, 1.40 NA objective. To ascertain sera specificity, pre-adsorption experiments using 5 µg of peptides were used together with labelling using an unrelated rabbit polyclonal antibody (data not shown). Donkey anti-rabbit was used without a primary antibody to make sure no cross reaction was to be observed (data not shown).

### Protein inhibitor experiment on salivary gland at 18 days post mosquito infection

Day 18 salivary gland sporozoites (SGS) were hand dissected from both wild type and *puf2-* parasite lines. 96-wells plate were seeded with 20,000 SGS in triplicate for both wild type and *puf2-* with or without 100 ug/ml (357.1 µM) of Cycloheximide (Sigma) in RPMI medium (without FBS supplement) and allowed to develop for 2 and 4 h at room temperature or 37°C. Three images were taken for each well using a widefield Zeiss Axiovert 200 M microscope, with 20x, 1.40 NA objective and parasites counted using ImageJ software to determine slender versus round.

### List of accession numbers

Puf1/UIS9 (PFE0935c, PBANKA_123350), Puf2 (PFD0825c, PBANKA_071920), Exp-2 (PBANKA_133430), Exp-1 (PBANKA_092670), Ama-1 (PBANKA_091500), GAP45 (PBANKA_143760), Myo-A (PBANKA_135570), Spect2 (PBANKA_100630), CelTOS (PBANKA_143230), Spect1 (PBANKA_135560), UIS4 (PBANKA_050120), UIS1/IK2 (PBANKA_020580), TLP1 (PBANKA_111600), TRSP (PBANKA_020910), SIAP1 (PBANKA_100620), MTRAP (PBANKA_051280), TREP (PBANKA_130650), PSOP9/GAMA (PBANKA_070190), P36p (PBANKA_100220), TFIIH (PBANKA_141340), RNA polymerase II subunit (PBANKA_020330), AP2 (PBANKA_083520 and PBANKA_010950), TFIIS Zinc-fingers (PBANKA_030420 and PBANKA_142110), Plasmepsin V (PBANKA_133870), RAD51 (PBANKA_093950), Histone H2B (PBANKA_094180) and ALBA3 (PBANKA_120440), Alveolin 9 (PBANKA_124060), Protein phosphatase 2C, putative (PBANKA_091340).

## Supporting Information

Figure S1 Sequence alignment and molecular model of *Plasmodium berghei* Puf2.
(TIF)

Figure S2 Generation and analysis of mutants lacking *puf1*.
(TIF)

Figure S3 Generation and analysis of mutants lacking expression of *puf2*.
(TIF)

Figure S4 Generation and analysis of mutant 1081cl1 lacking expression of *puf1* and *puf2*.
(TIF)

Figure S5 Development of mutant parasites in the mosquito.
(TIF)

Figure S6 *puf2*- (375 cl1) and *puf1-/2-* (1081 cl1) parasites transform into early EEFs in *Anopheles stephensi* mosquito salivary glands.
(TIF)

Figure S7 Microarray results for 11 genes initially tested by quantitative RT-PCR (see Figure 3).
(TIF)

Figure S8 Gene Ontology enrichment analysis clearly separates up-regulated transcripts from down-regulated ones.
(TIF)

Figure S9 Ultrastructure of *puf2*- salivary gland sporozoites on day 18 after *A. stephensi* mosquito infection.
(TIF)

Figure S10 30 days -old *puf2*- (375 cl1) and *eik2*- parasites do not transform into early EEFs in *A. stephensi* mosquito salivary glands to the same extend.
(JPG)

Table S1 Growth characteristics of blood and mosquito stage parasites of wild type, *puf1*- and *puf2*- parasites.
(DOC)

Table S2 Functionality and liver stage infectivity of *puf* gene deletion mutant.
(DOC)

Table S3 A-N. List of genes up- or down-regulated by microarray analysis using RMSANGER Affymetrix custom designed array.
(XLS)

Table S4 Details of the two *puf1*- *P. berghei* lines.
(DOC)

Table S5 Primers used for the generation and analysis of the *puf1*- lines.
(DOC)

Table S6 Details of the two *puf2*- lines.
(DOC)

Table S7 Primers used for the generation and analysis of *puf2*- lines.
(DOC)

Table S8 List of primer sequences used in RT-PCR and qRT-PCR experiments.
(DOC)

Video S1 Three-dimensional reconstruction of an *A. stephensi* salivary gland infected with wild type sporozoites.
(AVI)

Video S2 Three-dimensional reconstruction of an *A. stephensi* salivary gland infected with *puf1*- sporozoites.
(AVI)

Video S3 Three-dimensional reconstruction of an *A. stephensi* salivary gland infected with *puf2*- sporozoites.
(AVI)
