# Supplementary figures and images for: Transition of Plasmodium Sporozoites into Liver Stage-Like Forms Is Regulated by the RNA Binding Protein Pumilio
Source: PLoS Pathog. 2011 May 19;7(5):e1002046. doi: 10.1371/journal.ppat.1002046 (PMC3098293; doi:10.1371/journal.ppat.1002046)

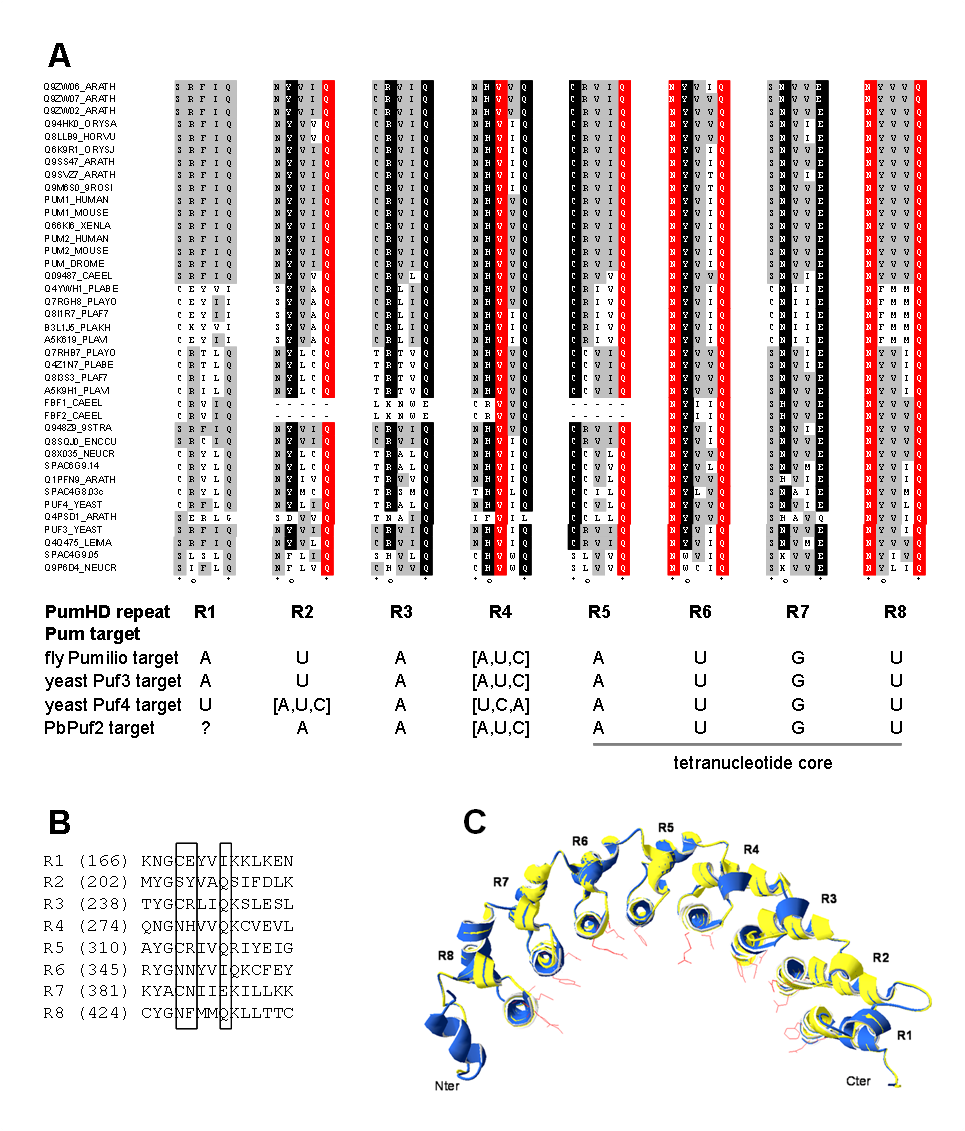

Supplement: Figure S1 — Sequence alignment and molecular model of Plasmodium berghei Puf2. (TIF) [file ppat.1002046.s001.tif]

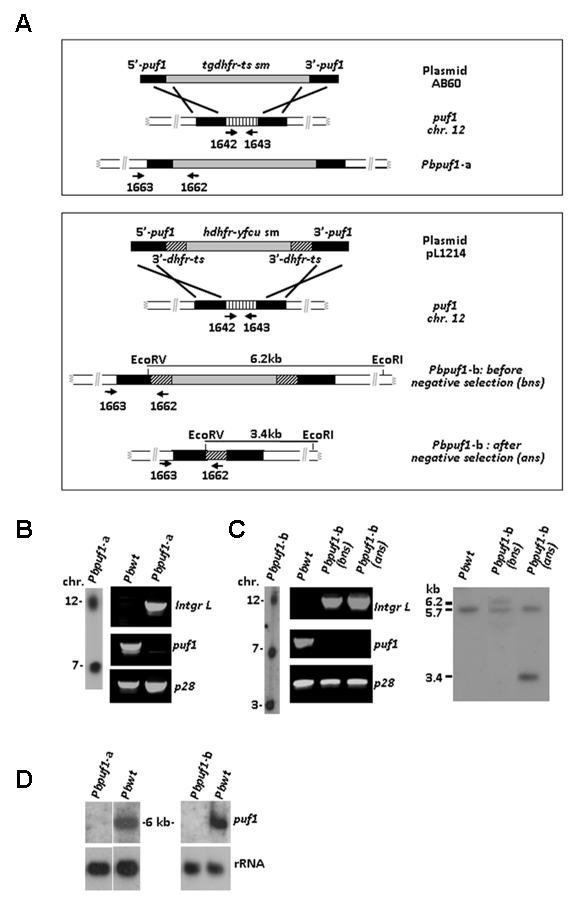

Supplement: Figure S2 — Generation and analysis of mutants lacking puf1. (TIF) [file ppat.1002046.s002.tif]

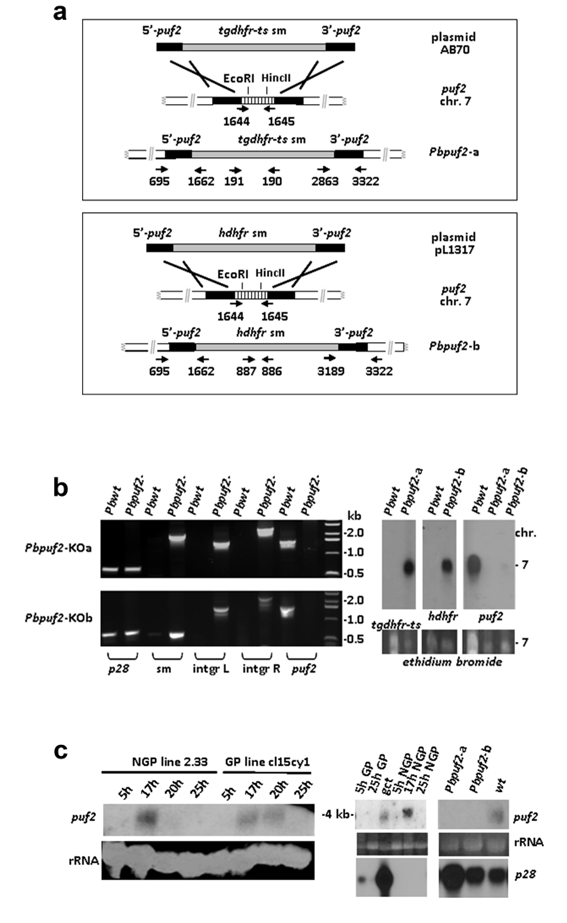

Supplement: Figure S3 — Generation and analysis of mutants lacking expression of puf2. (TIF) [file ppat.1002046.s003.tif]

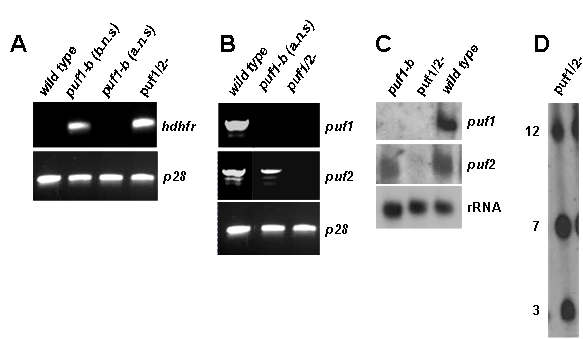

Supplement: Figure S4 — Generation and analysis of mutant 1081cl1 lacking expression of puf1 and puf2. (TIF) [file ppat.1002046.s004.tif]

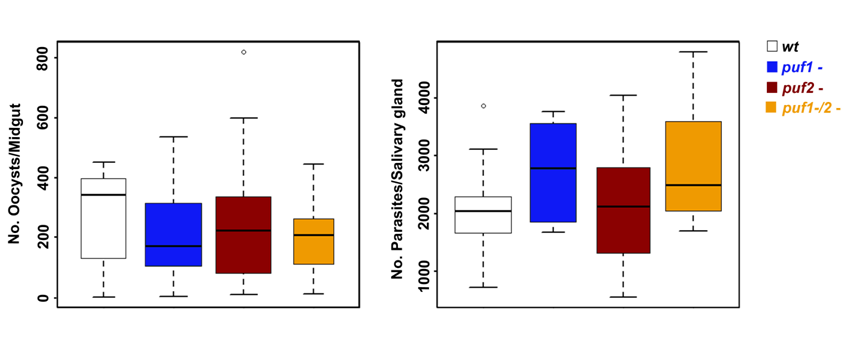

Supplement: Figure S5 — Development of mutant parasites in the mosquito. (TIF) [file ppat.1002046.s005.tif]

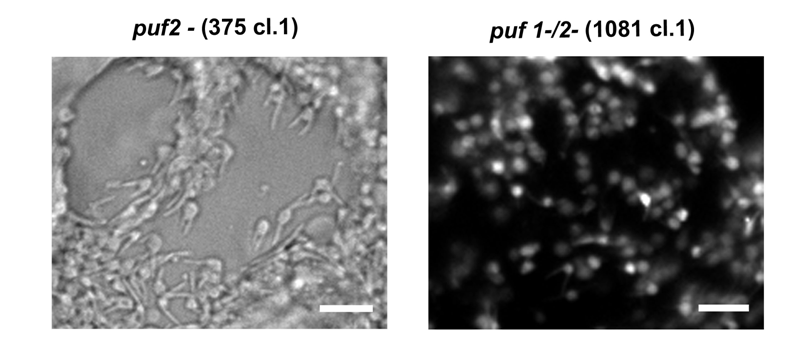

Supplement: Figure S6 — puf2- (375 cl1) and puf1-/2- (1081 cl1) parasites transform into early EEFs in Anopheles stephensi mosquito salivary glands. (TIF) [file ppat.1002046.s006.tif]

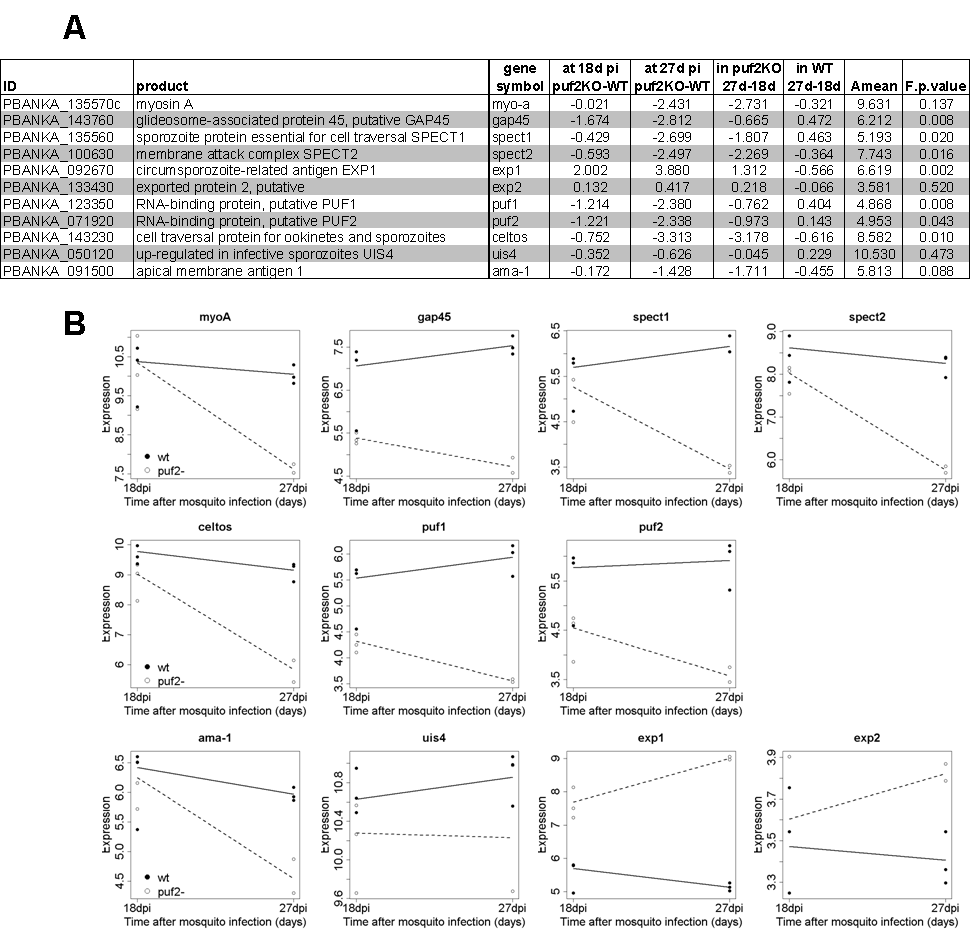

Supplement: Figure S7 — Microarray results for 11 genes initially tested by quantitative RT-PCR (see Figure 3). (TIF) [file ppat.1002046.s007.tif]

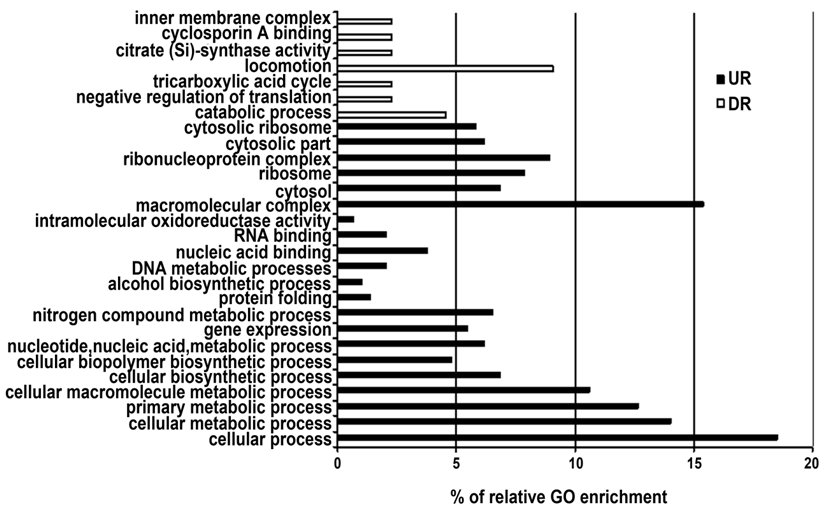

Supplement: Figure S8 — Gene Ontology enrichment analysis clearly separates up-regulated transcripts from down-regulated ones. (TIF) [file ppat.1002046.s008.tif]

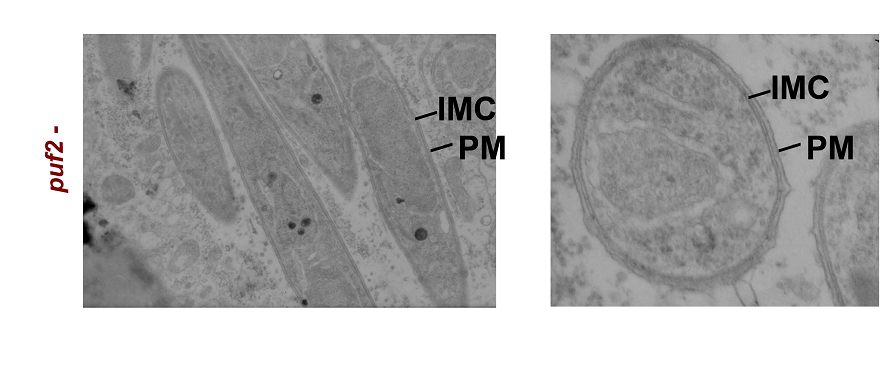

Supplement: Figure S9 — Ultrastructure of puf2- salivary gland sporozoites on day 18 after A. stephensi mosquito infection. (TIF) [file ppat.1002046.s009.tif]

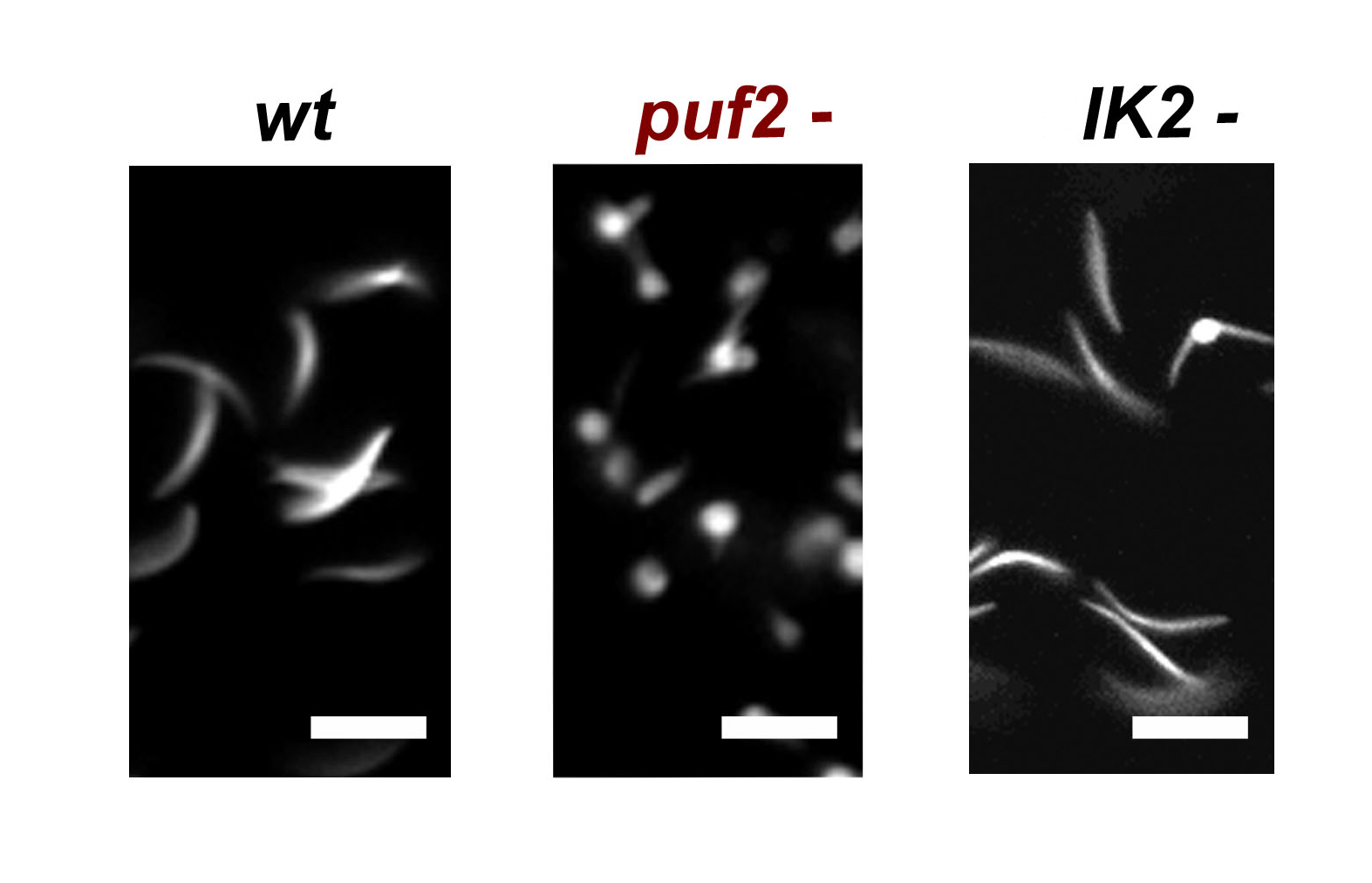

Supplement: Figure S10 — 30 days -old puf2- (375 cl1) and eik2- parasites do not transform into early EEFs in A. stephensi mosquito salivary glands to the same extend. (JPG) [file ppat.1002046.s010.jpg]
