# Supplementary material for: Transition of Plasmodium Sporozoites into Liver Stage-Like Forms Is Regulated by the RNA Binding Protein Pumilio
Source: PLoS Pathog. 2011 May 19;7(5):e1002046. doi: 10.1371/journal.ppat.1002046 (PMC3098293; doi:10.1371/journal.ppat.1002046)
Supplement: Table S1 — Growth characteristics of blood and mosquito stage parasites of wild type, puf1- and puf2- parasites. (DOC) [file ppat.1002046.s011.doc]

**Table S1. Growth characteristics of blood and mosquito stage parasites of wild type, *puf1*- and *puf2-* parasites.** The mean values and standard deviations (between brackets) are shown for the mutant lines. For the wild type parasites the range is shown of values obtained in at least 10 experiments.

|  | **clone** | **RMgm** | ***in vivo* multiplication rate ^1^** | **Gametocyte production (%) ^2^** | **Male gamete formation (%) ^3^** | **Female gamete formation (%) ^4^** | **Ookinete formation (%) ^5^** |
| --- | --- | --- | --- | --- | --- | --- | --- |
| *puf1*- | 351cl1 | RMgm513 | 8 (0) n=3 | nd | nd | nd | 62.4 (5.6) |
| *puf1*- | 900m2cl3 | RMgm514 | 8 (0) n=4 | 18.5 (2.6) | 79.3 (7.7) | 81.3 (3.0) | 60.0 (7.7) |
| *puf2*- | 375cl2 | RMgm515 | 8 (0) n=3 | 17.5 (1.3) | nd | nd | 70.0 (4.7) |
| *puf2*- | 1267cl2 | RMgm516 | 8 (0) n=4 | 20.3 (2.8) | 80.5 (8.2) | 79.3 (1.9) | 74.0 (3.9) |
| *puf1-/2-* | 1081cl1 | RMgm591 | 8 (0) n=4 | 18.8(2.8) | 83.8 (6.8) | 81.8 (6.2) | 61.3 (6.3) |
| wild type |  |  | 8 (0) n=8 | Range: 15-25 | Range: 75-95 | Range: 70-95 | Range: 50-90 |

^1^ The multiplication rate of asexual blood stages per 24 h was determined in mice infected with a single parasite. ^2^ Gametocyte production (gametocyte conversion rate) is the percentage of blood stage parasites that develop into gametocytes under standardized *in vivo* conditions. ^3^ Percentage of male gametocytes that emerge from the host cell and form gametes, determined by counting exflagellating male gametocytes. ^4^ Percentage of female gametocytes that emerge from the red blood cell and form gametes, determined by counting free female gametes. ^5^ Ookinete formation (ookinete conversion rate) is the percentage of female gametocytes that develop into mature ookinetes under standard *in vitro* assays for fertilization and ookinete development
