# Supplementary material for: Transition of Plasmodium Sporozoites into Liver Stage-Like Forms Is Regulated by the RNA Binding Protein Pumilio
Source: PLoS Pathog. 2011 May 19;7(5):e1002046. doi: 10.1371/journal.ppat.1002046 (PMC3098293; doi:10.1371/journal.ppat.1002046)
Supplement: Table S2 — Functionality and liver stage infectivity of puf gene deletion mutant. (DOC) [file ppat.1002046.s012.doc]

**Table S2. Functionality and liver stage infectivity of *puf* gene deletion mutants.** Each value represents the mean ± standard deviation from at least three independent experiments. Results are given as % of control. *puf2-* (375cl.1) experiments were performed once in order to confirm the results obtained with *puf2 -*(1267cl.2)**.** T-test * p ≤ 0.05, ** p ≤ 0.01; N.D., not determined

|  | ***puf1 -***  **(900m2cl3)** | ***puf2 -***  **(1267cl2)** | ***puf2 -***  **(375cl1)** | ***puf 1-/2 -***  **(1081cl1)** |
| --- | --- | --- | --- | --- |
| Gliding motility | 84±11% | N.D. | 28±5% | 18±3%** |
| Cell traversal | 133±12% | 25±2%* | N.D. | 55±10%** |
| Invasion | 104±2% | N.D. | 64±4%* | 51±1% |
| Development | 70±13% | 17±2%* | N.D. | 10±0.8%** |
| Parasite liver load | 34±18%** | 0.2±0%** | 0.3±0.3%* | 0.1±0.1%** |
