# Supplementary material for: Transition of Plasmodium Sporozoites into Liver Stage-Like Forms Is Regulated by the RNA Binding Protein Pumilio
Source: PLoS Pathog. 2011 May 19;7(5):e1002046. doi: 10.1371/journal.ppat.1002046 (PMC3098293; doi:10.1371/journal.ppat.1002046)
Supplement: Table S4 — Details of the two puf1- P. berghei lines. (DOC) [file ppat.1002046.s014.doc]

**Table S4. Details of the two *puf1- P. berghei* lines**

| Mutant | clone | Disrupted gene; genedb | Parent  parasite  line | parent  plasmid | primers  5’ targeting  region | primers  3’ targeting  region | plasmid |
| --- | --- | --- | --- | --- | --- | --- | --- |
|  |  |  |  |  |  |  |  |
| *puf1-* | 351cl1 | *puf1; PBANKA_123350* | cl15cy1 | pL0001 | 1514/1515 | 1522/1523 | AB60 |
| *puf1-* | 900m2cl3 | *puf1;* *PBANKA_123350* | 507cl1 (GFP+) | pL0035 | 1514/1515 | 2271/2272 | pL1214 |
