# Supplementary material for: Transition of Plasmodium Sporozoites into Liver Stage-Like Forms Is Regulated by the RNA Binding Protein Pumilio
Source: PLoS Pathog. 2011 May 19;7(5):e1002046. doi: 10.1371/journal.ppat.1002046 (PMC3098293; doi:10.1371/journal.ppat.1002046)
Supplement: Table S5 — Primers used for the generation and analysis of the puf1- lines. (DOC) [file ppat.1002046.s015.doc]

**Table S5. Primers used for the generation and analysis of the *puf1*- lines**

| No. | Primer sequences | region |
| --- | --- | --- |
|  | | |
| *Primers to amplify 5’ and 3’ target regions* | | |
| 1514 | AGGATCCGAACAAAACACATTGAAAATTCG | *5’ targeting region* |
| 1515 | AGATATCGTTCCTTGCATGTGTGTGTAG | *5’ targeting region* |
| 1522 | AAAGCTTGATATTGAAAAGCATGCTGTTC | *5’ targeting region* |
| 1523 | AAAGCTTAAGTTCATTGTAATCATAATGTT | *5’ targeting region* |
| 2271 | GGGCCCGTTAACGATATTGAAAAGCATGCTGTTC | *3’ targeting region* |
| 2272 | AAGCTTCCATGGTTAACTTCATTGTAATCATAATGTTG | *3’ targeting region* |
|  | | |
| *Primers for PCR analysis of puf1 disruption* | | |
| 537 | TGCTCTAGAATGAATTTTAAATACAGTTTTATT | *p28* |
| 538 | TGCTCTAGATTACATTACTATCACGTAAATAAC | *p28* |
| 1663 | ACTTTTTTTTGTGTGTGTGGG | 5’-*puf1*/ intgr L *∆puf1a/b* |
| 1662 | GATTCATAAATAGTTGGACTTG | AB60/pL1214/ intgr L *∆puf1a/b* |
| 1642 | CCATATGTTTAATGATAAAACTTTGATTAGC | puf1 |
| 1643 | GGGATCCTTATAATACTGGGTGTTTTTTTGCA | puf1 |
|  | | |
| Primers for generation of probes | | |
| 1642 | CCATATGTTTAATGATAAAACTTTGATTAGC | puf1 |
| 1643 | GGGATCCTTATAATACTGGGTGTTTTTTTGCA | puf1 |
| 692 | CGCGGATCCATGCATAAACCGGTGTGTC | *dhfr/ts* |
| 693 | CGCGGATCCGCTAGACAGCCATCTCCAT | *dhfr/ts* |
| L644R | GAACAAATTACTTCATTCATAGC | *large subunit ribosomal RNA* |
