# Supplementary material for: Transition of Plasmodium Sporozoites into Liver Stage-Like Forms Is Regulated by the RNA Binding Protein Pumilio
Source: PLoS Pathog. 2011 May 19;7(5):e1002046. doi: 10.1371/journal.ppat.1002046 (PMC3098293; doi:10.1371/journal.ppat.1002046)
Supplement: Table S6 — Details of the two puf2- lines. (DOC) [file ppat.1002046.s016.doc]

**Table S6. Details of the two *puf2*- lines**

| **Mutant** | **Clone** | **Disrupted gene** | **Parent**  **parasite**  **line** | **parent**  **plasmid** | **primers**  **5’ targeting**  **region** | **primers**  **3’ targeting**  **region** | **plasmid** |
| --- | --- | --- | --- | --- | --- | --- | --- |
|  |  |  |  |  |  |  |  |
| *puf2-* | 375cl1 | *puf2;* *PBANKA_071920* | cl15cy1 | pL0001 | 1544/1545 | 1546/1547 | AB70 |
| *puf2*- | 1267cl2 | *puf2; PBANKA_071920* | 507cl1 (GFP+) | pL0006 | 1544/1545 | 1546/1547 | pL1317 |
