# Supplementary material for: Transition of Plasmodium Sporozoites into Liver Stage-Like Forms Is Regulated by the RNA Binding Protein Pumilio
Source: PLoS Pathog. 2011 May 19;7(5):e1002046. doi: 10.1371/journal.ppat.1002046 (PMC3098293; doi:10.1371/journal.ppat.1002046)
Supplement: Table S7 — Primers used for the generation and analysis of puf2- lines. (DOC) [file ppat.1002046.s017.doc]

**Table S7. Primers used for the generation and analysis of *puf2*- lines**

| No. | Primer sequences | region |
| --- | --- | --- |
|  | | |
| *Primers to amplify 5’- and 3’- target regions* | | |
| 1544 | AGGATCCCCACATTCCATATACCC | 5’ targeting region |
| 1545 | TGATATCTTCTGTGTCATTTTCTTTAAG | 5’ targeting region |
| 1546 | TAAGCTTCCGAGTTTTTATATGCAAACA | 3’ targeting region |
| 1547 | TAAGCTTTCTAAATTTATGCAATTTAAAAAC | 3’ targeting region |
|  | | |
| *Primers for PCR analysis of puf2 disruption* | | |
| 537 | TGCTCTAGAATGAATTTTAAATACAGTTTTATT | *p28* |
| 538 | TGCTCTAGATTACATTACTATCACGTAAATAAC | *p28* |
| 190 | CGGGATCCATGCATAAACCGGTGTGTC | *tgdhfr-ts*/sm *∆puf2a* |
| 191 | CGGGATCCAAGCTTCTGTATTTCCGC | *tgdhfr-ts*/sm *∆puf2a* |
| 886 | GGAAGATCTATGGTTGGTTCGCTAAACTGCATCG | *hdhfr*/sm *∆puf2b* |
| 887 | GGAAGATCTTTAATCATTCTTCTCATATACTTC | *hdhfr*/sm *∆puf2b* |
| 695 | AATATTCATAACACACTTTTAAGC | 5’*puf2*/ intgr L *∆puf2a/b* |
| 1662 | GATTCATAAATAGTTGGACTTG | AB70/pL1317/ intgr L *∆puf2a/b* |
| 2863 | CGCATTATATGAGTTCATTTTAC | 5’*pbdhfr-ts*/ intgr R *∆puf2a* |
| 3189 | CTGGTGCTTTGAGGGGTG | 5’*eef1aa*/ intgr R *∆puf2b* |
| 3322 | CATTGGAAGCAAATTCTCTAATC | 3’*puf 2*/ intgr R *∆puf2a/b* |
| 1644 | CGGATCCTGATAATGCCATAATAAATGAAC | puf2 |
| 1645 | GGGATCCTCATGCCTCTAAATTATTAATAG | puf2 |
|  | | |
| Primers for generation of probes | | |
| 1644 | CGGATCCTGATAATGCCATAATAAATGAAC | puf2 |
| 1645 | GGGATCCTCATGCCTCTAAATTATTAATAG | puf2 |
| 741 | CGCGGATCCATGCATAAACCGGTGTGTC | *tgdhf/ts* |
| 742 | CGCGGATCCGCTAGACAGCCATCTCCAT | *tgdhfr/ts* |
| 886 | GGAAGATCTATGGTTGGTTCGCTAAACTGCATCG | *hdhfr* |
| 887 | GGAAGATCTTTAATCATTCTTCTCATATACTTC | *hdhfr* |
| 537 | TGCTCTAGAATGAATTTTAAATACAGTTTTATT | *p28* |
| 538 | TGCTCTAGATTACATTACTATCACGTAAATAAC | *p28* |
