# Supplementary material for: Transition of Plasmodium Sporozoites into Liver Stage-Like Forms Is Regulated by the RNA Binding Protein Pumilio
Source: PLoS Pathog. 2011 May 19;7(5):e1002046. doi: 10.1371/journal.ppat.1002046 (PMC3098293; doi:10.1371/journal.ppat.1002046)
Supplement: Table S8 — List of primer sequences used in RT-PCR and qRT-PCR experiments. (DOC) [file ppat.1002046.s018.doc]

**Table S8. List of primer sequences used in RT-PCR and qRT-PCR experiments.**

| **gene** | **genedb** | **fwd** | **sequence** | **rev** | **sequence** |
| --- | --- | --- | --- | --- | --- |
|  |  |  |  |  |  |
| *puf1* | *PBANKA_123350* | g0479 | ATAAGTGTTCATGGAACCCG | g0480 | TTACGCAGCACCCATGCC |
| *puf2* | *PBANKA_071920* | g0477 | GTTGACGACATTCCTGAGG | g0478 | TGCCTCTAAATTATTAATAGCCC |
|  |  |  |  |  |  |
| *spect-2* | *PBANKA_100630* | g0440 | AAGGAGTTTCAGCTATGCAC | g0441 | CAGTTCATTTATGCCTGACC |
| *spect* | *PBANKA_135560* | g0442 | TAGCCTAATTCAAATAAACGAAC | g0443 | GAAGTTAATTAATTCTGATACCCT |
| *celtos* | *PBANKA_143230* | g0454 | GTTCTATGTTTGAGAGGCAAAAATGG | g0455 | TGATGACGAGTCTTGTTGAAATGCAC |
| *gap45* | *PBANKA_143760* | g0153 | GTGGAGTAGTCTTTAAGG | g0154 | TATTGCAACAATTAAAGG |
| *myo-a* | *PBANKA_135570* | g0541 | ATGAATTAACTGAAAAGG | g0542 | TTGAACAATTCCAGTAGC |
| *exp-1* | *PBANKA_092670* | g0462 | AGGGAAGACATCCATTCCAAATTGG | g0463 | TGAAGATTTGGCATGTTAAGTGGTG |
| *exp-2* | *PBANKA_133430* | g0466 | ACGATCCAGGTTTGATTG | g0467 | TGGTAATAGTGGGACATTC |
| *uis4* | *PBANKA_050120* | g0444 | CCAAACCAAGCGATCATACATACAG | g0445 | CTTCACCCACTAAATCGCTTAATTC |
| *trap* | *PBANKA_134980* | g0432 | AACATTCACTCCATTCTTCC | g0433 | CATGTTATTCCAATGCTCAC |
|  |  |  |  |  |  |
| *18S rRNA* | *na* | PbA18SFw | AAGCATTAAATAAAGCGAATACATCCTTAC | PbA18SRev | GGAGATTGGTTTTGACGTTTATGTG |
| *mouse HPRT* | *na* | hprtfw | CATTATGCCGAGGATTTGGA | hprtrev | AATCCAGCAGGTCAGCAAAG |
